# In vivo study of dose-dependent antioxidant efficacy of functionalized core–shell yttrium oxide nanoparticles

**DOI:** 10.1007/s00210-022-02219-1

**Published:** 2022-02-24

**Authors:** Samr Kassem, Mahmoud M. Arafa, Manal M. Yehya, Mostafa A. M. Soliman

**Affiliations:** 1 Nanomaterials Research and Synthesis Unit, Animal Health Research Institute, ARC, Giza, Egypt; 2 Department of Biochemistry, Animal Health Research Institute, ARC, Giza, Egypt; 3 Department of Pathology, Animal Health Research Institute, ARC, Giza, Egypt; 4 Department of Byproduct Utilization, Animal Production Research Institute, ARC, Giza, Egypt

**Keywords:** Poly EGMP YNPs, Dose dependent, Antioxidant, Oxidative stress, Oxidative biomarkers, Blood–brain barrier, Neuroprotective

## Abstract

**Abstract:**

Herein, we assess the dose-dependent antioxidant efficacy of ultrafine spherical functionalized core–shell yttrium oxide nanoparticles (YNPs) with a mean size of 7–8 nm and modified with poly EGMP (ethylene glycol methacrylate phosphate) and N-Fluorescein Acrylamide. The antioxidant properties of these nanoparticles were investigated in three groups of Sprague–Dawley rats (10 per group) exposed to environmental stress daily for 1 week and one control group. Groups 2 and 3 were intravenously injected twice a week with YNPs at 0.3 and 0.5 mg at 2nd and 5th day of environmental stress exposure respectively. Different samples of blood and serum were collected from all experimental groups at end of the experiment to measure oxidative biomarkers such as total antioxidant capacity (TAC), hydroxyl radical antioxidant capacity (HORAC), oxygen radical antioxidant capacity (ORAC), malondialdehyde (MDA), and oxidants concentration as hydrogen peroxide (H_2_O_2_). The liver, brain, and spleen tissues were collected for fluorescence imaging and histopathological examination in addition to brain tissue examination by transmission electron microscope (TEM). Inductively coupled plasma-mass spectrometry (ICP-MS) was used to estimate YNPs translocation and concentration in tissues which is consecutively dependent on the dose of administration. Depending on all results, poly EGMP YNPs (poly EGMP yttrium oxide nanoparticles) can act as a potent direct antioxidant in a dose-dependent manner with good permeability through blood–brain barrier (BBB). Also, the neuroprotective effect of YNPs opening the door to a new therapeutic approach for modulating oxidative stress–related neural disorders.

**Highlights:**

• The dose-dependent antioxidant efficacy of ultrafine spherical functionalized core–shell yttrium oxide nanoparticles (YNPs) with a mean size of 7–8 nm and modified with poly EGMP (ethylene glycol methacrylate phosphate) and N-Fluorescein Acrylamide was assessed.

• The dose of administration directly affecting the brain, liver, and spleen tissues distribution, retention, and uptake of YNPs and direct correlation between the absorbed amount and higher dose administered.

• YNPs can act as a potent direct antioxidant in a dose-dependent manner with good permeability through blood–brain barrier (BBB).

**Graphical abstract:**

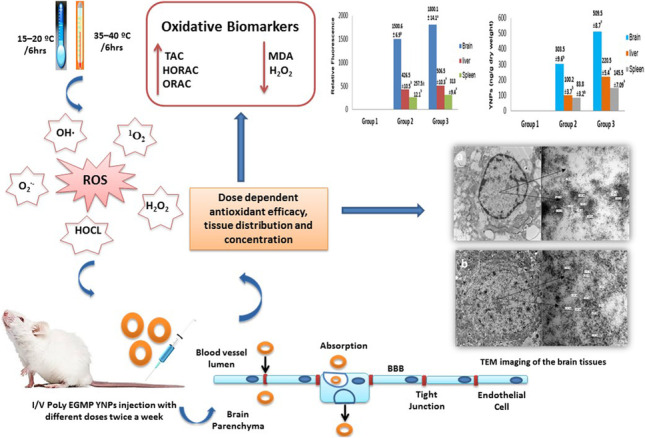

**Supplementary Information:**

The online version contains supplementary material available at 10.1007/s00210-022-02219-1.

## Introduction

The production of free radicals (ROS and RNS) surpasses the antioxidant systems’ ability to detoxify the generated reactive intermediates or repair cellular damage, resulting in oxidative stress (Di Meo et al. 2016; Zhang et al. 2016a, b, c). The generation of reactive oxygen species (ROS) during oxidative stress is critical for signal transduction and homeostasis (Ray et al. 2012). In general, ROS generation is a normal cellular function that is associated with various signaling pathways as well as the immune system’s defense mechanism. Superoxide anion (O_2_^**∙−**^), hydroxyl radical (OH^**∙**^), hydrogen peroxide (H_2_O_2_), singlet oxygen (^1^O_2_), and hypochlorous acid (HOCL) are all forms of reactive oxygen species (ROS). The reduction of a single electron catalyzed by the enzyme NADPH oxidase produces O_2_^**∙−**^, which is created from molecular oxygen (O_2_). Following the reduction of oxygen, either H_2_O_2_ is formed via dismutation or OH^**∙**^ is generated via the Fenton reaction (Thannickal and Fanburg 2002).

ROS are produced intrinsically in mitochondria through respiration, inflammatory responses, microsomes, and peroxisomes, whereas they are generated extrinsically by multiple stressors (Stowe and Camara 2009; Sarkar et al. 2014). Free radicals are released by inflammatory phagocytic cells like macrophages and neutrophils in exposure to microorganisms and environmental factors as a defensive mechanism (Risom et al. 2005). It is known, however, that when it is overproduced, it disrupts cellular membranes and biomolecules such as lipids, proteins, and DNA, resulting in adverse implications (Juan et al. 2021; Kumar and Pandey 2015), in addition oxidative stress–induced neurodegenerative disorders, including Creutzfeldt-Jakob disease, Bovine Spongiform Encephalopathy, Kuru, Gerstmann-Straussler-Scheinker syndrome, fatal familial insomnia, Parkinson’s disease (PD), Alzheimer’s disease (AD), and amyotrophic lateral sclerosis (ALS) (Dringen et al. 2005; Hritcu et al. 2008; Smith 2006; Sultana et al. 2008).

Stress can have a negative impact on health and, as a result, on productive and reproductive performance in commercial animals. Nutritional, industrial, environmental, and internal stressors have all been demonstrated to cause an excess of free radicals, a redox imbalance, and oxidative stress at the molecular level (Abuelo et al. 2019; Roth 2017). Oxidative stress has a number of adverse effects on the immunity and reproductive systems, as well as major growth, development, and health parameters in animals (Mavangira and Sordillo 2018; Sordillo 2016). Antioxidant enzymes and other non-enzymatic agents with the potential to diminish distinct chemical structures represent approximately the cellular antioxidant system, which is meant to prevent tissue damage. These compounds are mostly incapable of ensuring the balance between pro- and antioxidant agents. These compounds are mostly in capable of ensuring the balance between pro- and antioxidant agents and relieving oxidative stress (Kadiiska et al. 2013). Superoxide dismutase (SOD), catalase (CAT), glutathione peroxidase (GPx), and glutathione reductase (GR) are vital components of the enzymatic antioxidant defense mechanism, while non-enzymatic antioxidants include glutathione (GSH), thioredoxin (Trx), vitamins A, E, and C, flavonoids, trace elements, and proteins like albumin, ceruloplasmin, and metallothionein (Niedzielska et al. 2016).

Nanotechnology development has opened up new research areas focusing on diagnostic and therapeutic improvement. As a consequence, nanotechnology is being used to solve many of the problems related to treatment failure and disease progression. The potent antioxidant activity displayed by different nanomaterials has opened the door to promising possibilities for the development of innovative therapeutic strategies with enhanced efficacy (Singh et al. 2017; Soh et al. 2017). Nanomaterials have recently attracted a lot of attention for their potential application as nanomedicines. Antioxidants such as vitamins, trace minerals, and medicines have all been widely used to combat oxidative stress (Shokrzadeh et al. 2012; Lasram et al. 2014) but with a lot of limitations. Scientific contributions to this research area reveal that some NPs have a free radical scavenging activity and hence have proved effective in oxidative stress conditions (Caputo et al. 2014; Karakoti et al. 2012).

The larger ratio of surface area to volume found in nanoparticles, a feature that makes nanotechnology beneficial for catalysis, is responsible for some of the differences between the particles and bulk chemicals. Nanoparticles have a larger surface to volume ratio than bulk materials, which means they have greater surface area per unit volume for vacancy generation and elimination (Schubert et al. 2006). Although yttrium oxide nanoparticles (Y_2_O_3_ NPs) are widely applied in variable therapies and biological imaging due to their high luminescence efficiency (Zako et al. 2015), despite their free radical scavenging activity (Khaksar et al. 2017), Y_2_O_3_ NPs alone have received limited attention as a nanomedicine. Under oxidative stress, yttrium oxides may have antioxidant properties that elevate cells’ survival rate (Schubert et al. 2006). The free energy of generating yttrium oxide from elemental yttrium is one of the highest recorded (Kilbourn 1994). Under normal temperature and pressure, it is characterized by only mild deviation from stoichiometry, as well as the absorption of water and carbon dioxide from the environment (Kilbourn 1986). When compared to other inorganic nanoparticles, Y_2_O_3_ nanoparticles have unique characteristics such as a higher dielectric constant and better thermal stability (Rajakumar et al. 2021). Y_2_O_3_ is a prominent element that is used in biomedicine. Y_2_O_3_ has antibacterial applications (Kannan and Sundararajan 2015), cancer fighting (Nagajyothi et al. 2018), and used in the field of drug delivery system especially neurodegenerative disorders and hepatic failure (Schubert et al. 2006; Sönmez et al. 2015).

The blood-brain barrier (BBB) is a major roadblock in the delivery of drugs for neurodegenerative diseases. The BBB is one of the central nervous system’s most important defense mechanisms (CNS). Individual molecules, such as small lipid-soluble molecules, are allowed to flow through the capillary endothelial membrane whereas microorganisms and poisons are prevented (Zhou et al. 2018). However, during a disease state, this defense mechanism is a big barrier since it significantly reduces drug delivery. Various strategies, such as osmotic disruption of the BBB and chemical modification of active drug, have been used in recent years to help drugs cross the BBB. Furthermore, drug delivery using nanoparticles (NPs) is getting prominence as a non-invasive and effective way to treat cerebral disorders (Zhou et al. 2018). Non-invasiveness, low cost, good biodegradability and long-term sustainability, ease of synthesis, high targeting efficacy, and high reliability to load and deliver drugs across the BBB are only a few of the benefits of drug delivery with NPs (Nance et al. 2014; Zhang et al. 2016a, b, c).

In previous work, ultrafine YNPs were prepared and the surface modification was achieved by grafting a co-polymer made of poly EGMP to improve the biocompatibility and enhance biodistribution of YNPs with dose up to 0.1 mg/g bwt in normal 1-month-old Sprague-Dawley rats and pointed to the ability of NPs to distribute successfully to the brain shortly after tail vein injection without toxicity. Also, we approved that functionalized core-shell YNPs possess powerful antioxidant activity after one dose injection of 0.2 mg in a heat-stressed rat model but further research is needed to give answers on the dose-dependent antioxidant efficacy of YNPs with rescuing the oxidative stress–induced degenerative disorders in different organs especially the brain (Kassem et al. 2015, 2020; Sayour et al. 2019).

Our study aimed to investigate the dose-dependent antioxidant efficacy of ultrafine functionalized core-shell yttrium oxide nanoparticles (YNPs) with poly EGMP and N-Fluorescein Acrylamide as functionalized monomer to help in tracking YNPs in vivo. Our study is the first innovative study that investigates the ability of functionalized core-shell YNPs to pass through BBB which was approved by TEM imaging in an environmentally stressed rats model to focus on a new therapeutic approach for control oxidative stress–related neural disorders.

## Material and methods

### Synthesis of functionalized core–shell yttrium oxide nanoparticles

Ultrafine YNPs were synthesized as previously described (Kassem et al. 2015). After that, surface functionalization was performed by silanizing YNPs with 3-trimethoxysilylpropyl methacrylate and grafting an ethylene glycol methacrylate phosphate (EGMP) co-polymer, as described in Sayour et al. (2019) in addition to N-Fluorescein Acrylamide which was added in polymerization mixture as fluorescent monomer (Tan et al. 1992).

### Animals and experimental design

For this study, three groups of male Sprague–Dawley rats (groups 1–3), each consisted of ten animals and one control group that weighed 150 g, aged 1 month old at the time of experimentation, were obtained from VACSERA laboratories (Egypt) and acclimated for 7 days after arrival before study in the lab animal house, Animal Health Research Institute (Giza, Egypt). Rodents in three groups were housed in an animal room and exposed daily to different temperature degrees (35–40 °C) for 6 h per day and (15–20 °C) for 6 h per night for 1 week. Control group was housed in an animal room at a controlled temperature (21–24 °C), humidity (30–45%), and light cycle (12 h light/dark).To detect the antioxidant efficacy of poly EGMP yttrium oxide NPs, experimental groups 2 and 3 were injected intravenously via tail vein with YNPs at 0.3 and 0.5 mg respectively which dispersed in 300 µl of sterilized water for injection twice a week at 2nd and 5th day of environmental stress exposure. Experimental group 1 is the group exposed to the same stress conditions but without any treatment. At the end of the experiment, blood and serum samples were collected through cardiac puncture; also tissue sections from the liver, brain, and spleen were collected under Isoflurane anesthesia effect for histopathological evaluation and tracking of YNPs internalization by fluorescence microscope and tissue concentration by ICP-MS; in addition, brain sections were imaged by TEM to investigate the ability of YNPs to pass through BBB.

Food and water were provided ad libitum. The animal studies were approved by Research Ethics Committee for environmental and clinical studies (Protocol number: 181429) at Animal Health Research Institute (AHRI) and were carried out in accordance with Egyptian Ethics Committee Guidelines and the NIH Guidelines for the Care and Use of Laboratory Animals.

### Biochemical analysis of oxidative biomarkers

Oxidative stress was evaluated by measuring antioxidant activities as the total antioxidant capacity (TAC), hydroxyl radical antioxidant c(HORAC), and oxygen radical antioxidant capacity (ORAC), in addition oxidative byproduct level as malondialdehyde (MDA) and oxidants concentration as hydrogen peroxide (H_2_O_2_).

Blood samples were collected from all experimental groups at the end of the experiment without using an anticoagulant. Blood was left to clot for 30 min at 25 °C then the blood was centrifuged at 3000 rpm for 15 min at 4 °C. The top yellow serum layer was pipetted off without disturbing the white buffy layer. Serum samples were stored on ice and froze at − 80 °C till analysis. TAC was calorimetrically analyzed in 20 µl of serum samples based on the oxidation–reduction assay using test kits supplied by Biodiagnostics, Egypt, at a wavelength of 532 nm according to the method described by Koracevic et al. (2001). MDA level was calorimetrically measured in 200 µl of serum samples using test kits supplied by Biodiagnostics, Egypt, based on the reaction between MDA in the sample and thiobarbituric acid reagent at 95 °C for 30 min and the absorbance of the resultant pink product was measured at 534 nm (El Bana et al. 2016).

Plasma samples were collected from all experimental group’s blood at end of the experiment using heparin as an anticoagulant and centrifuged at 4 °C for 10 min. The plasma was removed and divided for testing. Blood plasma was diluted 100-fold or more with Assay Diluent before performing the following assays. HORAC assay was performed using a commercial assay kit (OxiSelect™ Hydroxyl Radical Antioxidant Capacity (HORAC) Activity Assay/STA-346–5, Cell Biolabs, Inc., San Diego, CA, USA). The HORAC activity assay is based on the hydroxyl radical oxidation of a fluorescent probe (fluorescein) through the hydrogen atom transfer (HAT) process (Zhang et al. 2021). The sample antioxidant capacity corresponding to the fluorescence decay curve and is used to measure total hydroxyl radical antioxidant activity in a sample and is evaluated by comparing to a gallic acid antioxidant standard curve at concentration ranged from 0 to 900 µM at an excitation wavelength of 480 nm and an emission wavelength of 530 nm. ORAC was performed using a commercial assay kit (OxiSelect™ Oxygen Radical Assay Kit (ORAC) Activity Assay/STA-343–5, Cell Biolabs, Inc., San Diego, CA, USA). The ORAC Activity Assay was performed by using a hydrogen atom transfer (HAT) technique to oxidize fluorescein as a fluorescent probe by peroxyl radicals. The sample antioxidant capacity corresponding to the fluorescence decay curve and is used to measure total peroxyl radical antioxidant activity in a sample and is evaluated by comparing to the water-soluble vitamin E analog Trolox standard curve at concentration ranged from 0 to 50 µM at an excitation wavelength of 485 nm and an emission wavelength of 520 nm (Xiao et al. 2021).

H_2_O_2_ assay kit was performed using a commercial assay kit (The OxiSelect™ Hydrogen Peroxide Assay Kit/STA-344, Cell Biolabs, Inc., San Diego, CA, USA). The assay kit is a sensitive quantitative fluorometric assay (Ansar et al. 2020). In the presence of horseradish peroxidase (HRP), non-fluorescent ADHP (10-Acetyl-3, 7 dihydroxy-phenoxazine) forms highly fluorescent Resorufin by reacting with H_2_O_2_ in a 1:1 stoichiometry which can be measured at an excitation of 530–560 nm and an emission of 590 nm by comparison with its respective standard curve of H_2_O_2_ at concentration ranged from 0 to 100 µM. The kit detection sensitivity limit was 50 nM (H_2_O_2_).

### Determination of YNPs internalization and tissue concentration

At the end of the experiment, frozen liver, brain, and spleen specimens were collected from experimental groups exposed to environmental stress and sectioned in a cryostat at 4-µm thickness, resolved in acetone for 5 min, rinsed in phosphate-buffered saline three times, and then placed on gelatin wrapped slides and observed under a fluorescent microscope (Olympus BX51) (Gal and Cagle 2005). Pixcavator IA standard edition software had been used to perform quantitative image analysis depending on intracellular densities of fluorescent markers which have been accurately determined (Li et al. 2011; Dimopoulos et al. 2014). At least, fluorescent intensity of each of randomly selected ten fluorescent images from each tissue specimens per each experimental group was scored and analyzed.

About of 1 g of brain, liver, and spleen tissues was mineralized in a microwave oven with high pressure then digested using mixture of concentrated nitric acid and 30% hydrogen peroxide; the prepared solution was diluted to 10 ml with deionized water in polypropylene vessels (Vidler et al. 2007). The concentration of YNPs in digested samples was quantitatively measured by ICP-MS instrument (Elan DRC-e, PerkinElmer, Germany).

### Histopathological studies

The liver, brain, and spleen specimens were collected from all experimental groups and preserved in 10% neutral buffered formalin and prepared for histopathological evaluation according to Suvarna et al. (2013).

### TEM imaging of YNPs in brain tissues

Brain tissue samples were sliced into around 1 mm of slices then prepared for TEM by fixation in glutaraldehyde and osmium tetroxide and processed according to Hunter et al. (1993); tissue grid was examined by transmission electron microscope JEOL (JEM-1400 TEM) and images were captured by CCD camera type AMT, optronic camera with 1632 × 1632 pixel size.

### Statistical analysis

ANOVA with Duncan multiple comparison tests was used to assess differences between three groups (duplicate per each group) using a statistical software program (SPSS for Windows, version 15, USA). All values are represented as mean ± standard error (SE) of 5 rats per replicate for each group.

## Results

### Biochemical analysis of oxidative biomarkers

**Fig. 1:**
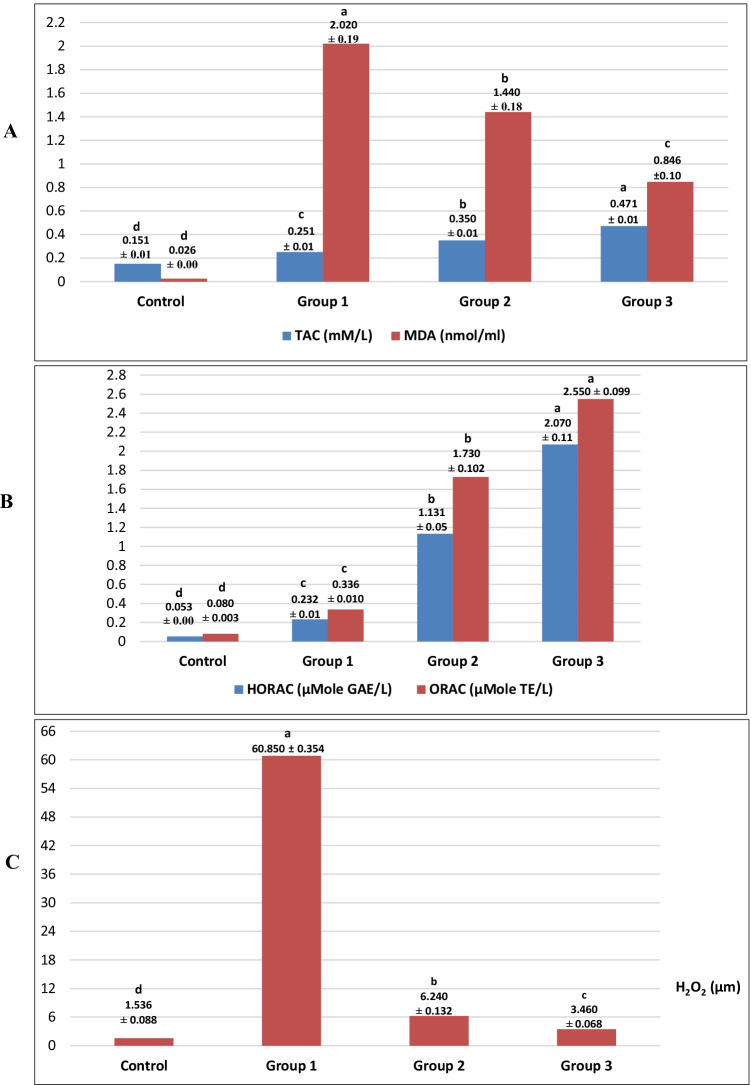
Biochemical profile of oxidative biomarkers of groups 2 and 3 compared with group 1. **A** Total antioxidant capacity (TAC) and malondialdehyde (MDA). **B** Hydroxyl radical antioxidant capacity (HORAC) and oxygen radical antioxidant capacity (ORAC). **C** Hydrogen peroxide (H_2_O_2_). Values with different superscripts (a, b, and c) in the same row are statistically significantly (P < 0.05) different between the experimental groups. All values are represented as mean ± SE

Oxidative stress was successfully induced in all experimental groups (1, 2, and 3) by daily exposure to different temperature degrees (35–40 °C) for 6 h per day and (15–20 °C) for 6 h per night for 1 week compared with control one (Fig. 1A–C) and supplementary file 4. The antioxidant activity of poly EGMP yttrium oxide NPs was evaluated by measuring variable oxidative biomarkers post twice a week injection of YNPs at 0.3 and 0.5 mg at 2nd and 5th day of environmental stress exposure for experimental groups 2 and 3 respectively compared with group 1 which did not receive any treatment. Our study revealed a significant increase (*P* < 0.05) in TAC concentration and a significant decrease in MDA concentration (Fig. 1A) in experimental groups 2 and 3 compared with mean values of group 1.

Figure 1B reveals a significant increase (*P* < 0.05) in HORAC and ORAC concentrations in experimental groups 2 and 3 compared with mean values of group 1. On the other hand, a significant decrease (*P* < 0.05) in H_2_O_2_ concentration (Fig. 1C) was recorded in experimental groups 2 and 3 compared with group 1.

### YNPs internalization and tissue concentration

**Fig. 2:**
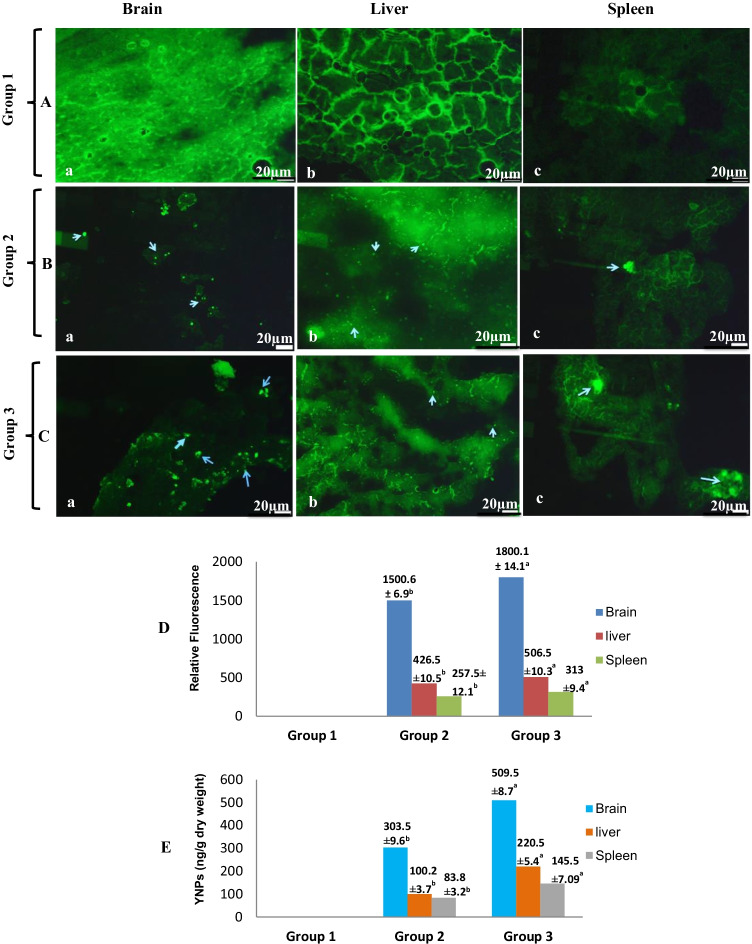
YNPs internalization and tissue concentration. **A**–**C** Fluorescence microscope images of the brain, liver, and spleen tissues at 1 week after a twice doses of injection of YNPs derived from different groups. **A** Images of group 1, showing (a–c) clearance of all tissues without fluorescein-labeled nanoparticles. **B** Images of group 2 which was injected with twice doses of 0.3 mg YNPs, showing (a) high internalization and distribution of fluorescein-labeled YNPs in the brain tissue and mild internalization in the liver tissue (b) and spleen tissue (c). **C** Images of group 3 which was injected with twice doses of 0.5 mg YNPs, showing (a) high internalization and distribution of fluorescein-labeled YNPs in the brain tissue and moderate internalization in the liver tissue (b) and spleen tissues (c). **D** Histogram of YNPs internalization and distribution in the brain, liver, and spleen tissues at end of the experiment after double doses of injection of YNPs per week for 1 week derived from all environmentally stressed groups. **E** YNPs concentration in various tissues derived from all environmentally stressed groups at end of the experiment by ICP-MS. Values with different superscripts (a and b) in the same row are statistically significantly (*P* < 0.05) different between the experimental groups. All values are represented as mean ± SE

Cerebral localization and distribution of fluorescence-labeled YNPs in the brain and internalization in the liver and spleen tissues were detected by fluorescence microscope as green dense particles as shown in Fig. 2A–D.

Figure 2A is fluorescence imaging of group 1, showing (a–c) clearance of all tissues without fluorescein-labeled nanoparticles. Figure 2B of group 2 which was injected with twice doses of 0.3 mg YNPs for 1 week shows (a) high cerebral localization and distribution of fluorescein-labeled YNPs in the brain tissue and mild internalization in the liver tissue (b) and spleen tissue (c). Figure 2C of group 3 which was injected with twice doses of 0.5 mg YNPs for 1 week shows (a) high cerebral localization and distribution of fluorescein-labeled YNPs in the brain tissue and moderate internalization in the liver tissue (b) and spleen tissues (c). Figure 2D reveals quantitative internalization of YNPs and distribution in the brain, liver, and spleen tissues at end of the experiment derived from experimental groups 1, 2, and 3 depending on quantitative image analysis of fluorescent intensity. Significance of relative fluorescence (*P* < 0.05) was showed in brain tissue followed by liver and spleen tissues in group 2 and 3 in dose-dependent manner compared with group 1.

Tissue concentration of YNPs was detected using ICP-MS as shown in Fig. 2E, and the highest significant concentration (ng/g dry weight) (*P* < 0.05) was observed in the brain followed by the liver then spleen tissues of group 2 and group 3 at dose-dependent manner compared with group 1.

### Histopathological studies

**Fig. 3:**
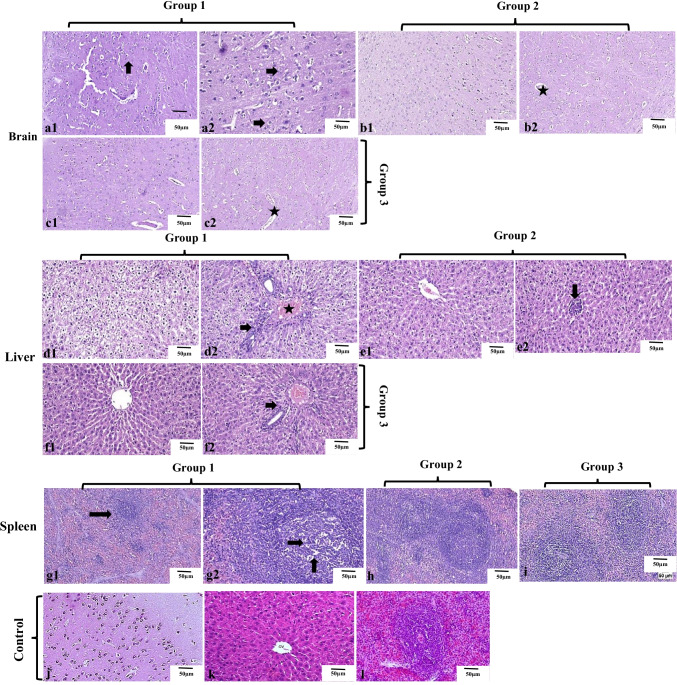
Histopathological examination of the brain, liver, and spleen tissues derived from all experimental groups. (a1 and a2) Images of group 1 brain sections, showing (a1) perineural edema with gliosis in the striatum (arrow), (a2) perivascular edema with mild astrocytosis (arrows) in the cerebral cortex. (b1 and b2) Images of group 2 brain sections which was injected with twice doses of 0.3 mg YNPs per week for 1 week, showing (b1) apparently normal cerebral cortex, (b2) mild perineural edema in the striatum (star). (c1 and c2) Images of group 3 brain sections which was injected with twice doses of 0.5 mg YNPs per week for 1 week, showing (c1) apparently normal cerebral cortex, (c2) very mild perineural edema in the striatum (star). (d1 and d2) Images of group 1 liver sections, showing (d1) excessive vacuolated hepatocytes, (d2) congested blood vessels (star) in the portal area with few mononuclear inflammatory cells infiltration (arrow). (e1 and e2) Images of group 2 liver sections, showing (e1) apparently normal hepatocytes surrounding the central vein, (e2) mild focal mononuclear cell infiltration (arrow). (f1 and f2) Images of group 3 liver sections, showing (f1) apparently normal hepatic cords radiating from the central vein, (f2) minute mononuclear cells were detected in the portal area (arrow). (g1 and g2) Images of group 1 spleen sections, showing (g1) relatively small lymphoid follicle (arrow) with expansion of the red pulp, (g2) lymphoid depletion with lymphocytolysis (arrows). (h and i) Images of group 2 and 3 spleen sections respectively showing apparently normal splenic tissue. (j–l) images of control group brain, liver (with central vein cv), and spleen sections with normal histological architecture

Histopathological examination of the brain, liver, and spleen tissues derived from all experimental groups is shown in Fig. 3. Microscopic examination of the brain tissue from group 1 (Fig. 3a1 and a2) revealed mild gliosis in the cerebral cortex with marked distension of the perivascular spaces. The striatum showed gliosis along with edema. Regarding the brain tissues from group 2 (Fig. 3b1 and b2), the cerebral cortex appeared apparently normal, but the striatum showed mild edema. Likewise, an apparently normal cerebral cortex was observed in group 3 (Fig. 3c1 and c2) with very mild perineural edema at the striatum.

Microscopic examination of liver tissue from group 1 revealed severe and diffuse vacuolation of hepatocytes in which the cells were swollen with clear cytoplasm. Aggregations of mononuclear inflammatory cells were infiltrating the hepatic parenchyma as well as the portal areas. Perivascular edema was commonly noticed in some portal areas (Fig. 3d1 and d2). Concerning group 2 (Fig. 3e1 and e2), mild to moderate hepatocellular vacuolation was noticed that was associated with few infiltrations of inflammatory cells in some circumstances. Group 3 (Fig. 3f1 and f2) showed apparently normal hepatic parenchyma in several examined sections. 

Histopathological examination of the spleen from group 1 (Fig. 3g1 and g2) showed relatively smaller lymphoid aggregates with a great expansion of the red pulp. The lymphoid follicles showed mild depletion with lymphocytolysis manifested by fragmented lymphocytes. On contrary, splenic tissue from group 2 (Fig. 3h) appeared apparently normal with average-sized follicles and numerous lymphoid cells. Similarly, an apparently normal spleen was noticed in tissues from group 3 (Fig. 3i) that appeared free from any detectible alterations. Histopathological examination of the control group (Fig. 3j–l) showed normal histological architecture of brain, liver, and spleen sections. Further histopathological findings of the brain, liver, and spleen tissues were represented in supplementary files 1, 2, and 3 respectively.

### TEM imaging of YNPs in brain tissues

**Fig. 4:**
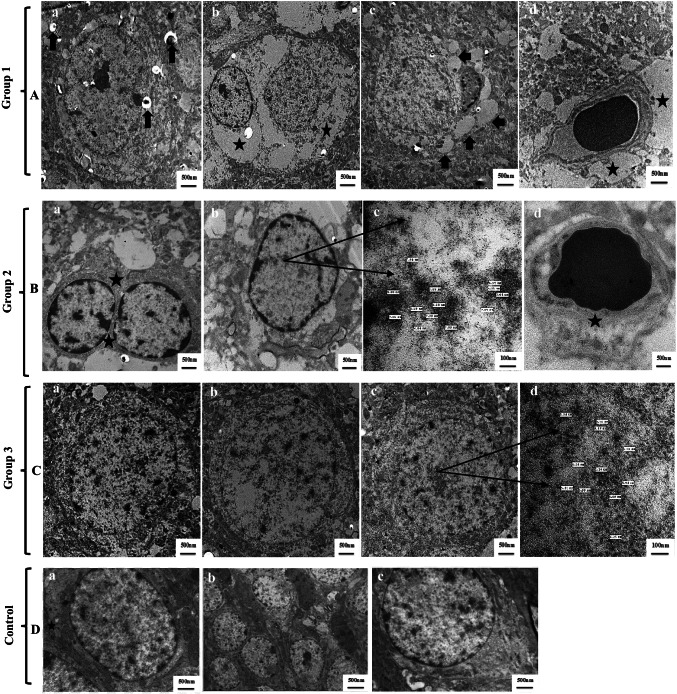
Transmission electron microscope images of the brain tissues derived from all experimental groups. **A** Brain tissue images of group 1, showing (a) marked vacuolization of cytoplasm (arrows), (b) astrocyte with cytoplasmic swelling and severe neural edema (stars), (c) swelling of mitochondria and the endoplasmic reticulum was expanded (arrows), and (d) regions of swelling surrounding the capillaries (stars). **B** Brain tissue images of group 2 which was injected with twice doses of 0.3 mg YNPs, showing (a) reduced levels of edema in the neurons (stars) and (d) in the area surrounding the capillaries (star), (b and c) limited cytoplasmic vacuolization and intracellular localization of ultrafine YNPs ranged 5–7 nm in size (arrow) as electron-dense particles. **C** Brain tissue images of group 3 which was injected with twice doses of 0.5 mg YNPs, showing (a and b) apparently normal brain structure with the disappearance of neural edema and cytoplasmic vacuolization, (c and d) intracellular localization of ultrafine electron-dense YNPs ranged 4–6 nm in size (arrow) in the cytoplasm. **D** Brain tissue ultrastructure of control group, showing (a) normal tree-like dendritic appearance of neurons (star), (b and c) neuronal cell has clear boundaries with continuous and definite plasma membrane

Transmission electron microscope imaging of the brain tissues derived from all experimental groups is shown in Fig. 4A–C. TEM examination of the brain tissue from group 1 (Fig. 4A (a–d)) revealed marked vacuolization of cytoplasm, astrocyte with cytoplasmic swelling and severe neural edema, swelling of mitochondria and the endoplasmic reticulum was expanded, and regions of swelling surrounding the capillaries. Regarding TEM imaging of the brain tissue derived from group 2 (Fig. 4B (a–d)), reduced levels of edema detected in the neurons and the area surrounding the capillaries with limited cytoplasmic vacuolization and intracellular localization of electron-dense ultrafine YNPs ranged 5–7 nm in size. On the other hand, apparently normal brain structure with the disappearance of neural edema and cytoplasmic vacuolization was observed in TEM imaging of the brain tissue derived from group 3 (Fig. 4C (a–d)) with intracellular localization of ultrafine YNPs ranged 4–6 nm in size as electron-dense particles. TEM imaging of the brain tissue from the control group (Fig. 4D (a–c)) showed normal neuronal appearance with clear boundaries and well definitive plasma membrane.

## Discussion

This study investigates the dose-dependent antioxidant efficacy of ultrafine functionalized yttrium oxide nanoparticles (YNPs) with poly EGMP in an environmentally stressed Sprague–Dawley rats model. Experimental groups (1, 2, and 3) were daily exposed to different temperature degrees (35–40 °C) for 6 h per day and (15–20 °C) for 6 h per night for 1 week which resulted in oxidative stress induction. Oxidative biomarkers as TAC, MDA, HORAC, ORAC, and H_2_O_2_ levels were measured post twice a week injection of YNPs at 0.3 and 0.5 mg at 2nd and 5th day of environmental stress exposure for experimental groups 2 and 3 respectively to detect the dose-dependent antioxidant efficacy. Our study revealed a significant increase (*P* < 0.05) in TAC, HORAC, and ORAC concentrations with a significant decrease (*P* < 0.05) in MDA and H_2_O_2_ concentration (Fig. 1A–C) in experimental groups 2 and 3 compared with group 1. As a result, our study noticed the potent antioxidant activity of YNPs in a dose-dependent manner evidenced by increasing antioxidant potency against hydroxyl and oxygen radicals as well as reduction in hydrogen peroxide concentration, and rescuing the ROS damage effect on lipid layers in cell membrane resulted in a decrease in MDA concentration in environmentally stressed groups injected by YNPs compared with non-injected one.

Yttrium oxide is an inorganic rare earth element in the lanthanide series and has many valence states. It is surrounded by an oxide lattice. Yttrium is a one-of-a-kind antioxidant with a lanthanide-like electron configuration due to its atomic features. In its stable, oxide form, yttrium sustains a + 3 oxidation state (Mitra et al. 2014). YNPs have the unique property of showing little deviation from stoichiometry after absorbing water and carbon dioxide at normal temperatures and pressures (Schubert et al. 2006). Depending on the YNPs structure and the unique nature of their size range specially ranged from 6 to 1000 nm, Y_2_O_3_ NPs can protect cells from oxidative stress–induced cell death (Mitra et al. 2014). YNPs have been shown to be able to reduce the cascade of non-NP oxides that are not protective, revealing size and structure considerations for nanoparticle antioxidant efficiency (Schubert et al. 2006). The antioxidant activities of YNPs were thoroughly investigated as a free radical scavenger (Tavoosi et al. 2020). The fact that YNPs protect against oxidative stress has a variety of explanations. They may behave as direct antioxidants, restrict ROS production by inhibiting apoptosis, or cause a low level of ROS production that triggers the initiation of a ROS defense system before the glutamate-induced cell death stage is completed.

The latter is a type of pretreatment that can occur when cells were exposed to particulates that is known to produce lower levels of ROS (Schubert et al. 2006; Becker et al. 2002). Moreover, YNPs have a function comparable to enzymes like CAT and SOD, and can remain active in cellular level as an essential factor. In fact, YNPs can interfere with cell defense mechanisms such as apoptosis and rescue cells from apoptosis by modulating biological processes; additionally, they have enzymatic activity similar to SOD (Tavoosi et al. 2020). The therapy with YNPs reduced oxidative stress biomarkers and inflammatory cytokines in a dose-dependent manner (Khurana et al. 2019). As reported by Schubert et al. (2006), in the HT22 hippocampal nerve cell line, Y_2_O_3_ nanoparticles revealed a direct dose-dependent antioxidant effect; 20 µg/mL of Y_2_O_3_ particles reduced generated ROS by around 50%, and 200 µg/mL of Y_2_O_3_ particles reduced accumulated ROS by tenfold. As a result, the protective reaction must be based on the nanoparticle’s physico-chemical properties, which are generally independent of its size and are most definitely Redox properties (Schubert et al. 2006).

N-Fluorescein Acrylamide was added in polymerization mixture as fluorescent monomer during surface functionalization of poly EGMP YNPs to enhance the fluorescent intensity and help in tracking YNPs in vivo. Cerebral localization and distribution of fluorescence-labeled YNPs in the brain, internalization in the liver and spleen tissues, and YNPs concentration in variable tissues derived from experimental groups exposed to environmental stress were investigated (Fig. 2A–E) by fluorescence microscope imaging as a rapid, simple, and low-cost technique. Internalization and distribution of fluorescein-labeled YNPs in the different tissues were found to be in a dose-dependent way. Our study revealed significant localization (*P* < 0.05) of positively charged spherical YNPs in the brain tissues more than liver and spleen tissues of environmentally stressed groups injected by YNPs compared with non-injected one depending on the dose of injected nanoparticles as showed in Fig. 2D. The concentration of YNPs in the brain, liver, and spleen tissues was investigated using ICP-MS which revealed that the significant highest concentration (*P* < 0.05) of particles was observed in the brain followed by the liver then spleen in group 3 compared with group 2 at dose-dependent way. It was expected that the measured concentration of YNPs include the tissues and blood vessels as well, and the biodistribution of particles was completely judged by fluorescence microscope imaging and TEM, especially of brain tissues.

These findings approved the theory that the dose is one of the most important factors affecting tissue distribution, retention, and uptake of NPs and direct correlation between the absorbed amount and higher dose administered (Kumari et al. 2014). TEM analysis of brain tissue is necessary to give proof of localization of YNPs either in blood vessels, endothelial cells of micro vessels or brain parenchyma. Figure 4B and C approve passage of ultrafine spherical poly EGMP YNPs to the BBB (Fig. 4B (b, c) with a size of 5–7 nm and Fig. 4C (c, d) with a size of 4–6 nm compared with control group).

Surface modification of nanoparticles by coating them with biocompatible surfactant or biodegradable polymers like poly EGMP in our study, or covalent ligation with targeted ligands, has been proven to be an effective technique to improve their penetrating performance and permeability. For successful BBB penetration, it delays opsonization and extends the circulation duration of NPs in the blood stream (Li and Sabliov 2013). Because of the delayed reticuloendothelial absorption of injected nanoparticles due to the greatly extended surface coating, nanoparticles have a higher possibility of circulating in the blood for a longer period and then passing through brain tissues (Yokel et al. 2013).

The BBB, constituted by the brain microvascular system, is a membrane barrier in the central nervous system (CNS) of most species, including animals, avian, and mammals that separates blood from the extracellular fluids of the brain (Mayer et al. 2009; Cserr and Bundgaard 1984). The BBB protects the brain from toxic substances in the bloodstream and maintains a regular brain environment (Harati et al. 2012). The most basic compositional parts are astrocyte end feet, basement membrane, and tight junctions between endothelial cells. They are in charge of controlling blood flow in the brain capillaries by contracting and relaxing (Jespersen and Østergaard 2012). Through efflux pumps such as ATP-binding cassette transporters, additional barrier processes can actively restrict the amount of medicines transported to the brain parenchyma. As a result, these intrinsic cellular features make the BBB a problematic biological barrier obstacle to drug delivery into the brain.

The mechanisms of endocytosis and cellular uptake of nanoparticles in trials linked to drug delivery systems across the BBB can be classified into passive diffusion (Sela et al. 2015), carrier-mediated transport (Zhang et al. 2016a, b, c), receptor-mediated (Clark and Davis 2015), and absorption-mediated endocytosis (Cheng et al. 2014) depending on the physico-chemical properties of nanoparticles as size, shape, surface charge, and functional groups on the surface and chemical composition of nanoparticles. The size of the nanoparticles is a key factor that influenced the endocytic mechanism and cellular uptake. In general, nanoparticles with diameters of less than 200 nm are internalized predominantly through the clathrin-mediated endocytic mechanism, whereas larger particles around 500 nm are internalized through the caveolae-mediated mechanism (Hillaireau and Couvreur 2009). Particles with a diameter of more than 500 nm can rapidly accumulate in the liver and spleen, but particles with a diameter less than 5 nm are filtered by the kidneys and eliminated (Blanco et al. 2015).

Because of their higher electrostatic interactions with the negatively charged plasma membrane, positive surface charge nanoparticles are typically internalized to a greater extent by cells than negatively charged ones (Hirsch et al. 2013; Hühn et al. 2013). Furthermore, it has been reported that spherically shaped nanoparticles penetrate cells more readily than non-spherically shaped particles, which could be due to the unique curvature of the adsorbed nanoparticles experienced by the cell (Florez et al. 2012). YNPs have shown ROS potential scavenger behavior to be able to protect cells against oxidative stress born damage effect. Our microscopic findings of the brain, liver, and spleen tissues derived from all experimental groups approved the ability of YNPs to rescue cells from oxidative stress–induced cellular deterioration depending on dose of administration in experimental groups.

Microscopic examination of liver tissue concerning YNPs injected groups 2 and 3 with twice doses of 0.3 and 0.5 mg respectively. Group 2 (Fig. 3e1 and e2) revealed mild to moderate hepatic lesions as hepatocellular vacuolation with few inflammatory cell infiltrations and group 3 (Fig. 3f1 and f2) appeared apparently normal hepatic parenchyma in different sections compared with group 1 which exposed to the same environmental stress conditions without YNPs injection that revealed severe hepatic lesions and mononuclear inflammatory cells aggregations (Fig. 3d1 and d2). Song et al. (2019) recently demonstrated that intraperitoneal injection of 30 mg/kg of Y_2_O_3_ NPs with spherical morphology and size range of 40–100 nm into lipopolysaccharide-induced rats enhanced hepatic antioxidant status and reduced oxidative stress and inflammatory responses. They also discovered that Y2O3 NPs decreased hepatic NF-B (nuclear factor-kappa B) activation, apoptosis, and liver damage, suggesting that they could be used as a novel therapeutic method for fulminant hepatic failure treatment. Furthermore, it is efficient in oxidative stress–related disorders. Hosseini et al. (2013) demonstrated that yttrium oxide nanoparticles combination with cerium oxide significantly exhibited antioxidant properties controlling cell apoptosis. Moreover, histopathological findings of the spleen from group 2 (Fig. 3h) and group 3 (Fig. 3i) appeared apparently normal with average-sized follicles and numerous lymphoid cells to free from any detectible lesions in both groups respectively compared with splenic lesions recorded in group 1 (Fig. 3g1 and g2) which enhance the hypothesis of potent direct antioxidant activity of YNPs in most of the sensitive tissues. It was hypothesized that in vitro, YNPs inhibited lipopolysaccharide-induced oxidative damage and in macrophages, it reduced ROS, superoxide radical release, and recovered mitochondrial membrane potential (Khurana et al. 2019).

Light microscopic examination (Fig. 3a–c) is complementary with TEM imaging (Fig. 4A–C) of the brain tissues derived from all experimental groups. Light microscopic examination of the brain tissues of groups 2 and 3 (Fig. 3b–c) showed improved neuropathological lesions in a dose-dependent manner with mild to very mild findings regarding perineural edema, while group 1 had neural damage with marked edema in the cerebral cortex and vacuolated cytoplasm (Fig. 3a). TEM imaging is helpful in ensuring intracellular internalization of ultrafine poly EGMP YNPs in the brain parenchyma and successful passage through BBB. In addition, investigating the therapeutic effect and reducing oxidative stress born neural damage at the cellular level. Schubert et al. (2006) discussed the effects of Y_2_O_3_ nanoparticles on nerve cells, as well as their protection from oxidative stress lethality and neuroprotection to be independent of particle size but almost on dose and physico-chemical properties. Yttrium oxide can act as a direct antioxidant, controlling or neutralizing the ROS required to destroy the cells. As a result, Y_2_O_3_ has proven to be a potent antioxidant and neuroprotective against oxidative stress and programmed cell death. As a result, it can be concluded that Y_2_O_3_ nanoparticles can assist neuronal cell survival against oxidative stress, which could be relevant in therapeutic applications (Schubert et al. 2006).

## Conclusion

Our study successes to focus on the dose-dependent antioxidant activity of novel functionalized core–shell YNPs in vivo. Furthermore, tracking of fluorescein-labeled YNPs in different tissues such as the brain, liver, and spleen is an easy, low-cost tool of detection. Poly EGMP YNPs not only able to pass through the BBB which is approved by fluorescence and TEM imaging but also help in rescuing the different cells from oxidative stress–related damage. Histopathological examination of the liver and spleen sections collected from experimental groups 2 and 3 which were intravenously injected twice a week with YNPs at 0.3 and 0.5 mg at 2nd and 5th day of environmental stress exposure respectively revealed improved hepatic and splenic antioxidant status with moderate to mild lesions compared with group 1 which did not receive any treatment. TEM imaging of the brain tissue is comparable with histopathological findings which revealed the neuroprotection effect of YNPs in vivo which resulted mainly from ROS blocking as a direct antioxidant activity together with physico-chemical properties especially surface functionalization.

Taken together with biochemical analysis of oxidative biomarkers, these results prove that poly EGMP YNPs can act as therapeutic regenerative particles that possess excellent dose-dependent antioxidant capabilities and able to pass through BBB with rescuing the enhancing neuroprotection and hepatic antioxidant performance. Our findings add to the information of YNPs’ ability to modulate oxidative stress–related neural disorders. However, more research is needed to ensure that YNPs have a therapeutic effect in neurodegenerative models.

## Supplementary Information

Below is the link to the electronic supplementary material.

Supplementary file1 (PDF 2234 KB)

Supplementary file2 (PDF 1999 KB)

Supplementary file3 (PDF 3210 KB)

Supplementary file4 (XLSX 11 KB)

## Data Availability

All raw data and graphs are available upon request.
