# Supplementary material for: In vivo study of dose-dependent antioxidant efficacy of functionalized core–shell yttrium oxide nanoparticles
Source: Naunyn Schmiedebergs Arch Pharmacol. 2022 Feb 24;395(5):593–606. doi: 10.1007/s00210-022-02219-1 (PMC8989852; doi:10.1007/s00210-022-02219-1)
Supplement: Supplementary file 1 — Supplementary file1 (PDF 2234 KB) [file 210_2022_2219_MOESM1_ESM.pdf]

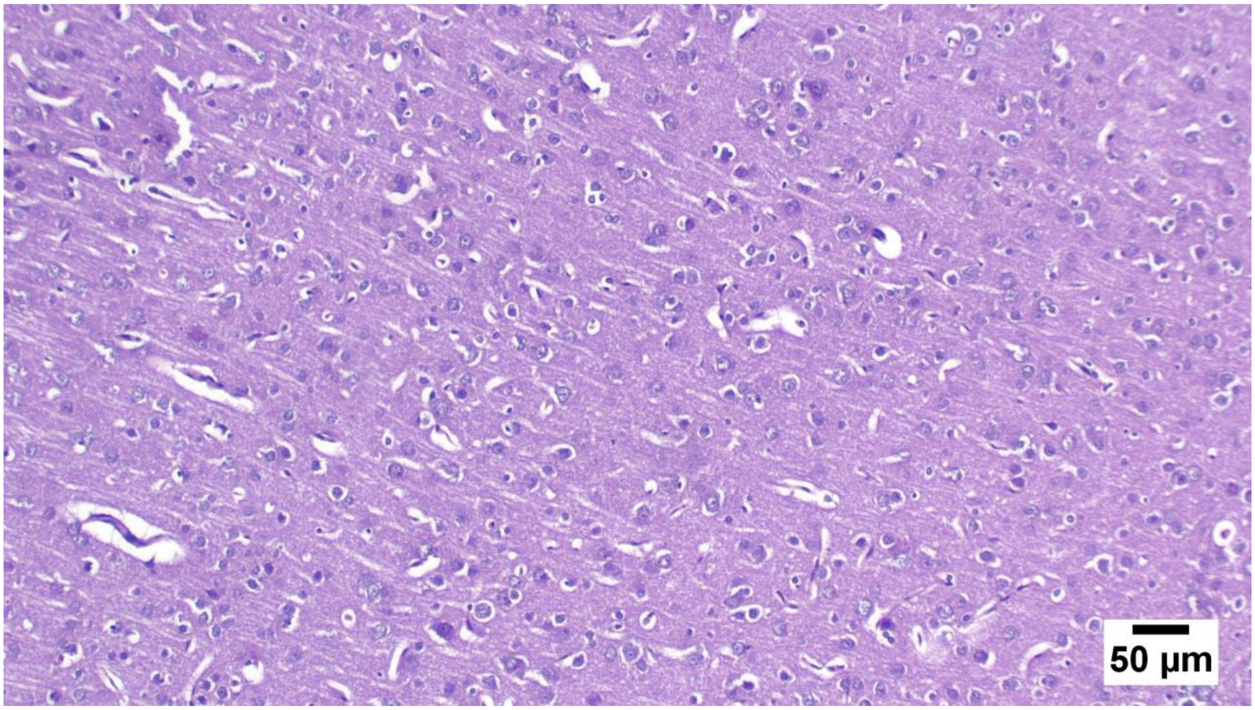

Photomicrograph of brain cerebral cortex, group 1 showing mild gliosis in the cerebral cortex (H&E).

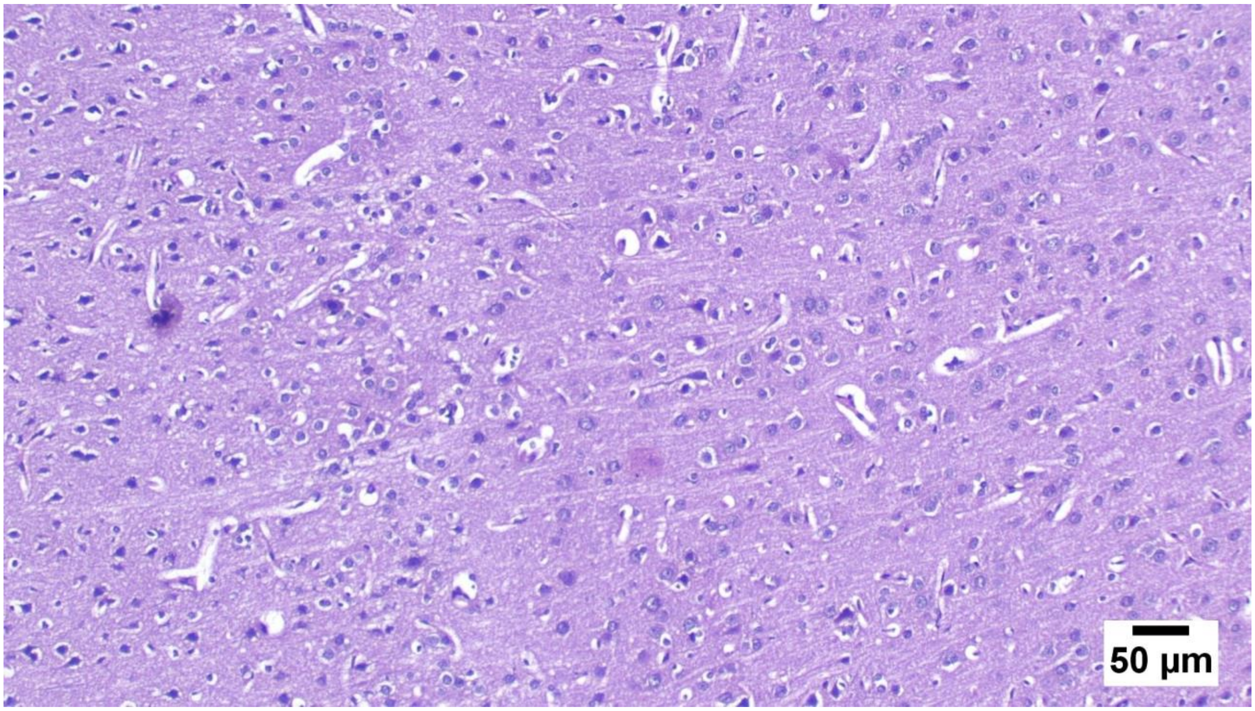

Photomicrograph of brain cerebral cortex of group 1 showing mild gliosis in the cerebral cortex (H&E).

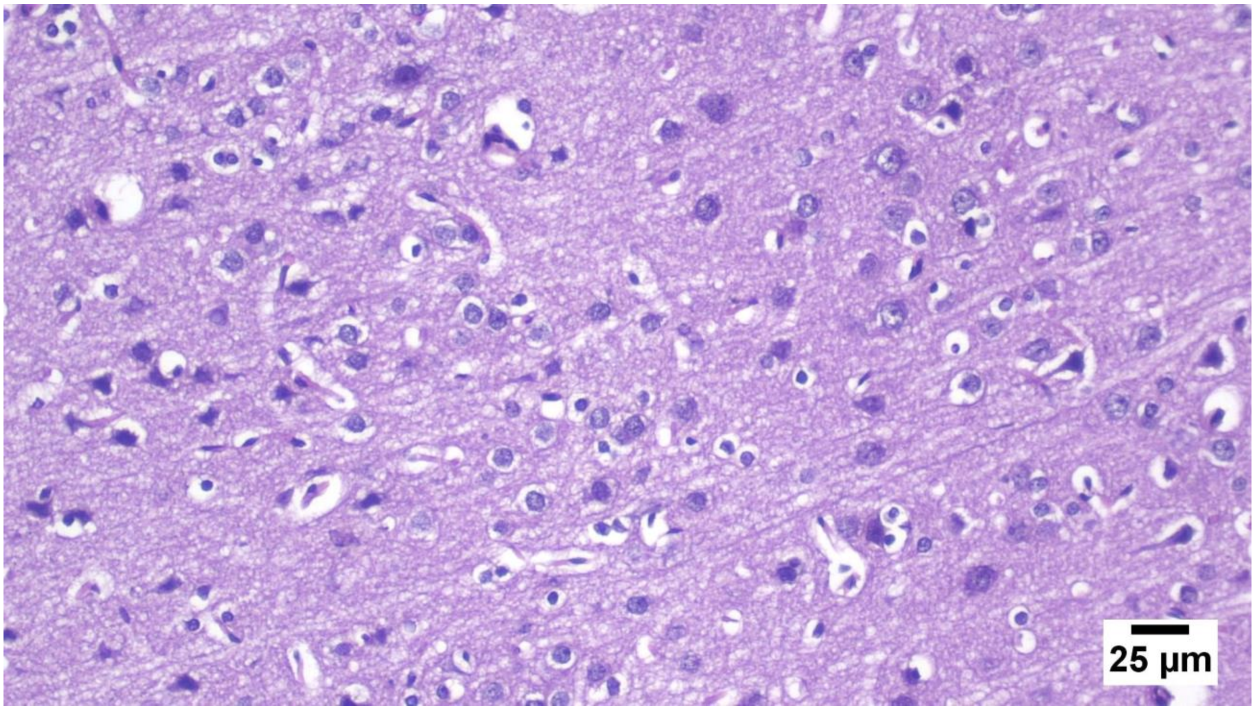

Photomicrograph of brain cerebral cortex, group 1 showing neuronal edema (H&E).

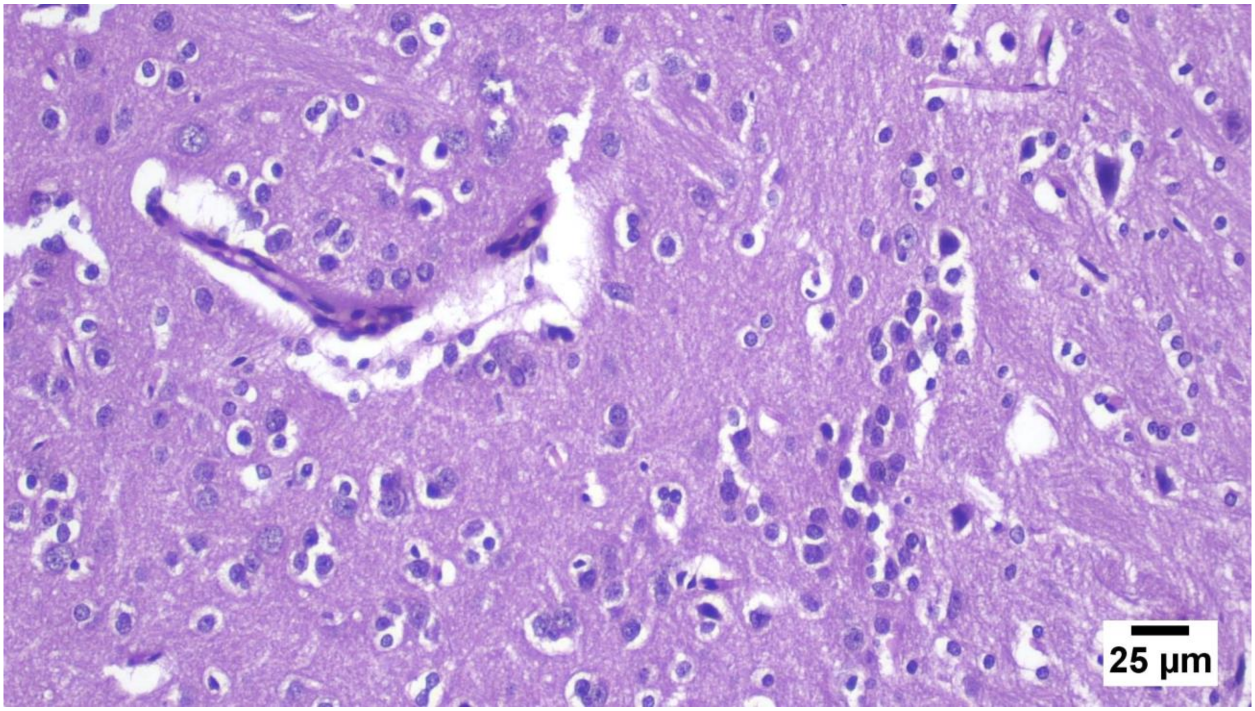

Photomicrograph of brain striatum, group 1 showing neuronal and perivascular edema with gliosis (H&E).

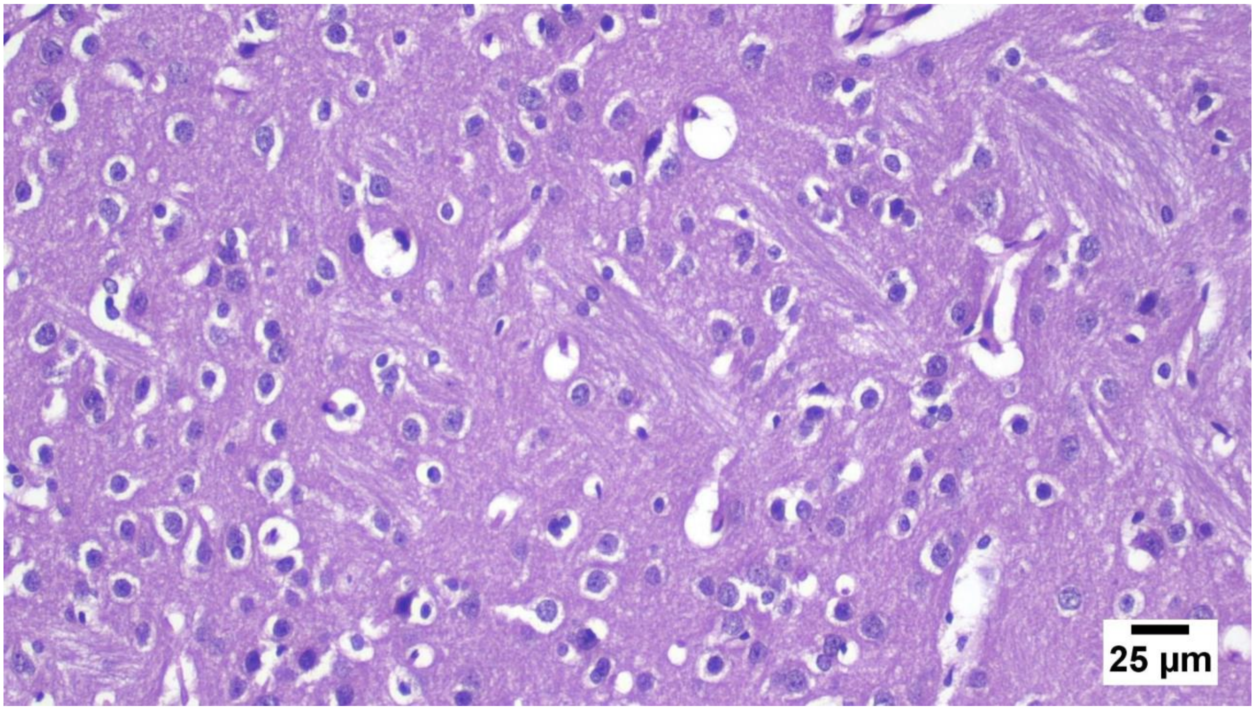

Photomicrograph of brain striatum of group 1 showing neuronal edema (H&E).

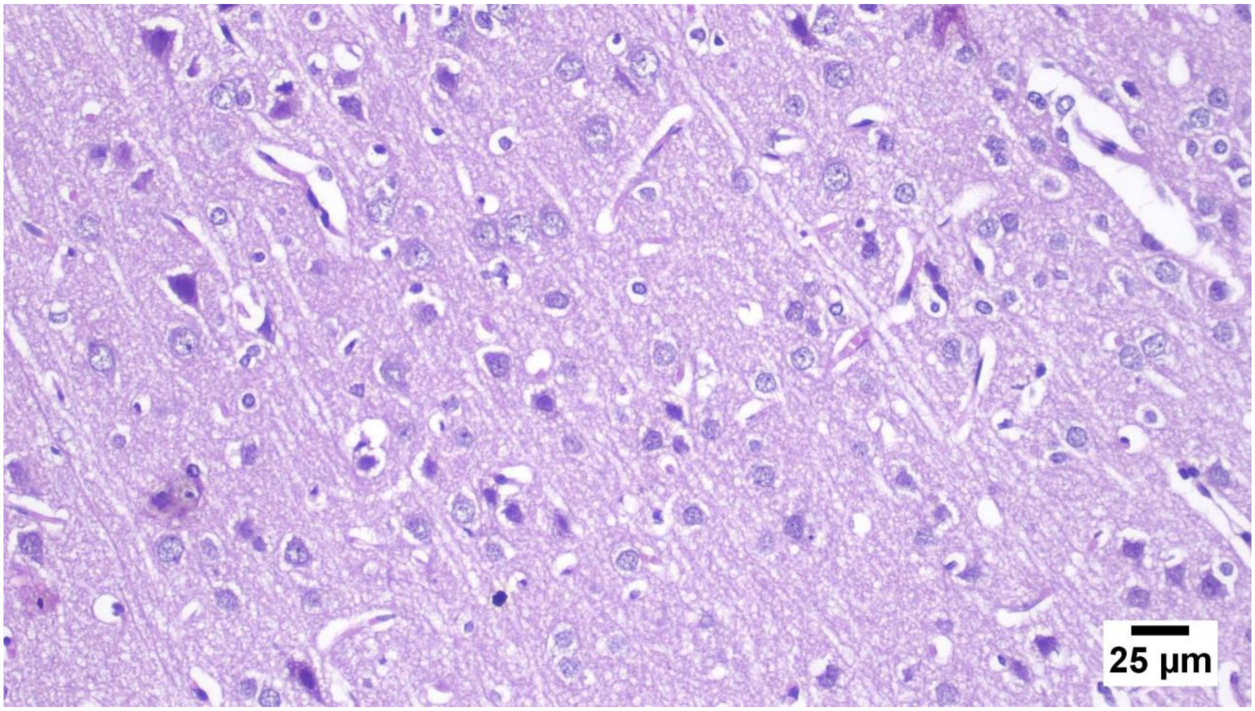

Photomicrograph of brain cerebral cortex of group 2 showed higher magnification showing apparently normal cerebral cortex (H&E).

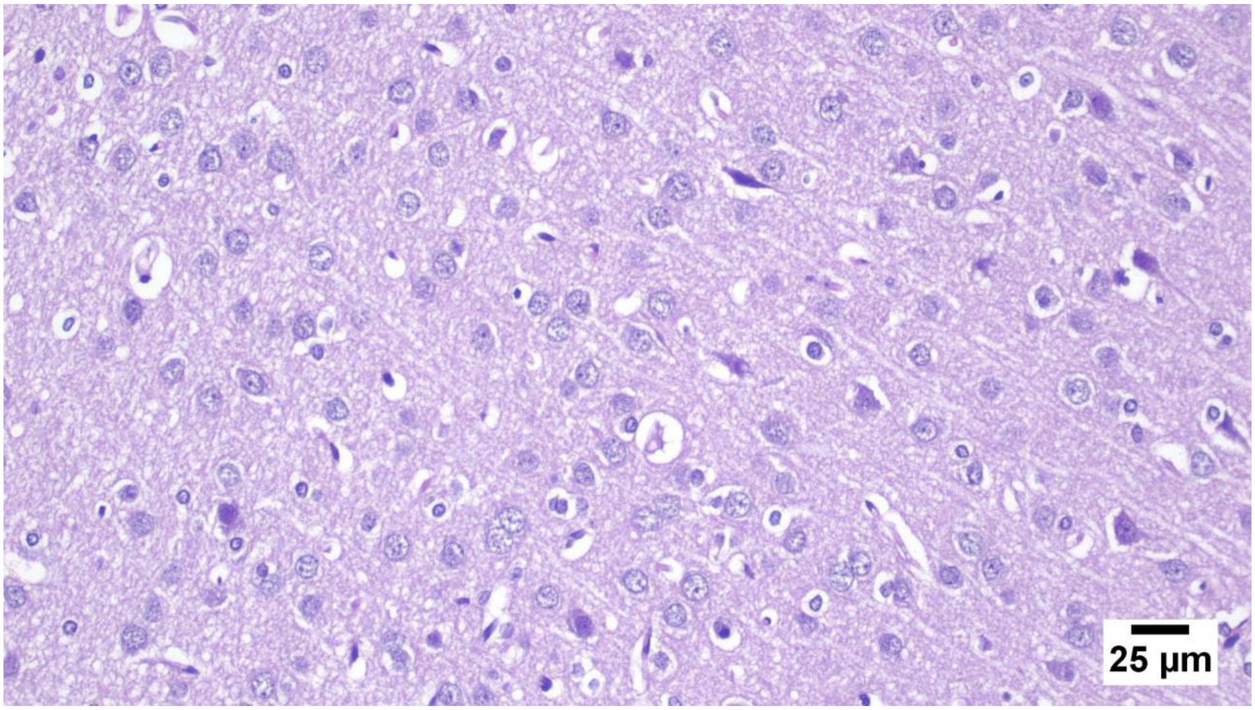

Photomicrograph of brain cerebral cortex of group 2 showed higher magnification showing apparently normal cerebral cortex (H&E).

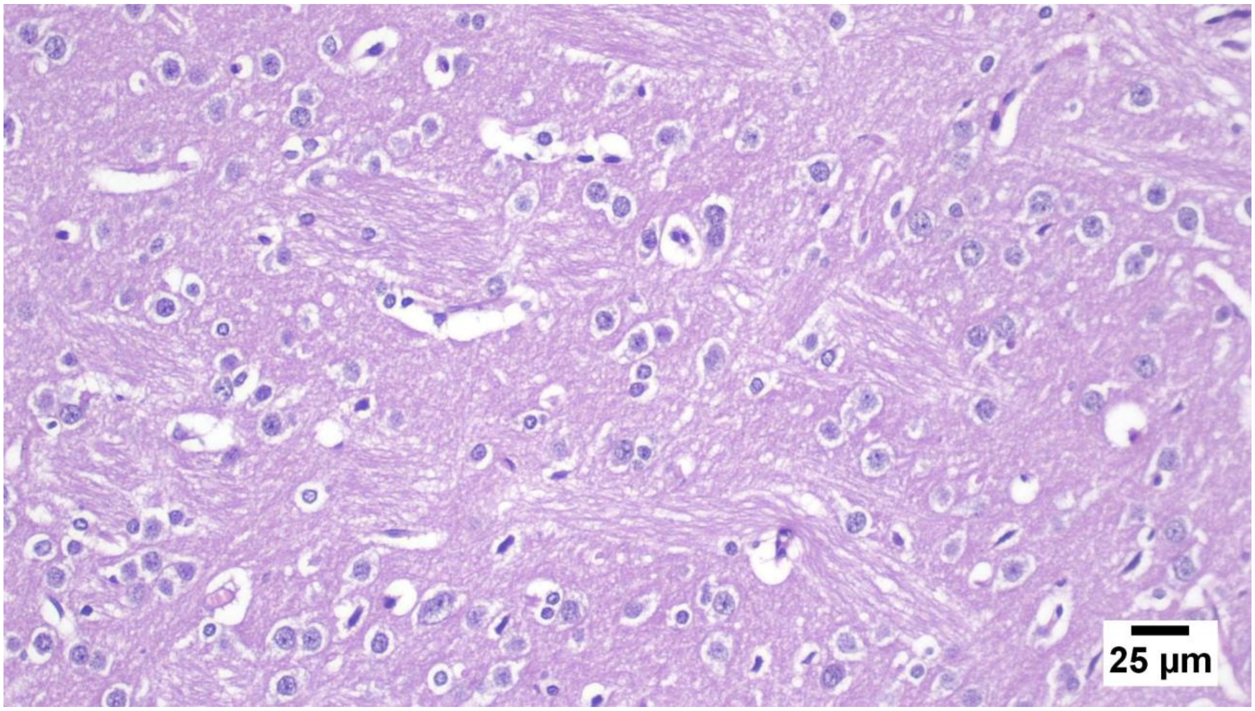

Photomicrograph of brain striatum of group 2 showed higher magnification showing mild edema (H&E).

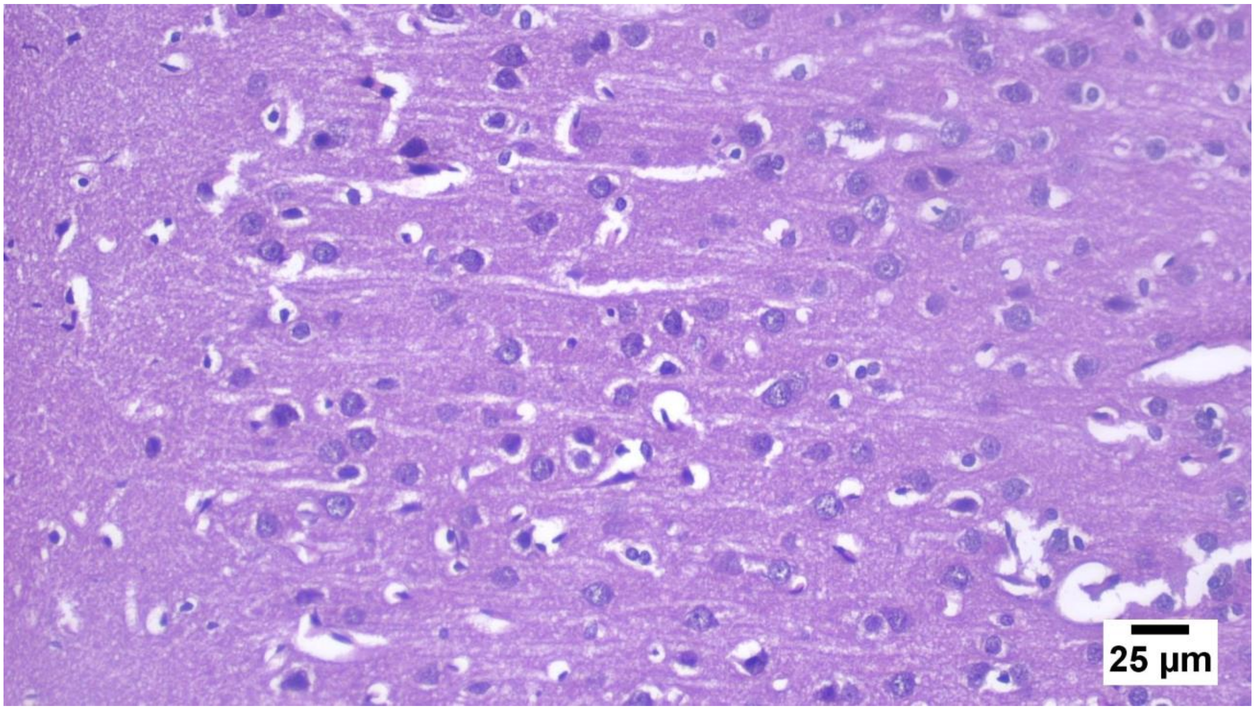

Photomicrograph of brain cerebral cortex, group 3 showed higher magnification showing apparently normal cerebral cortex (H&E).

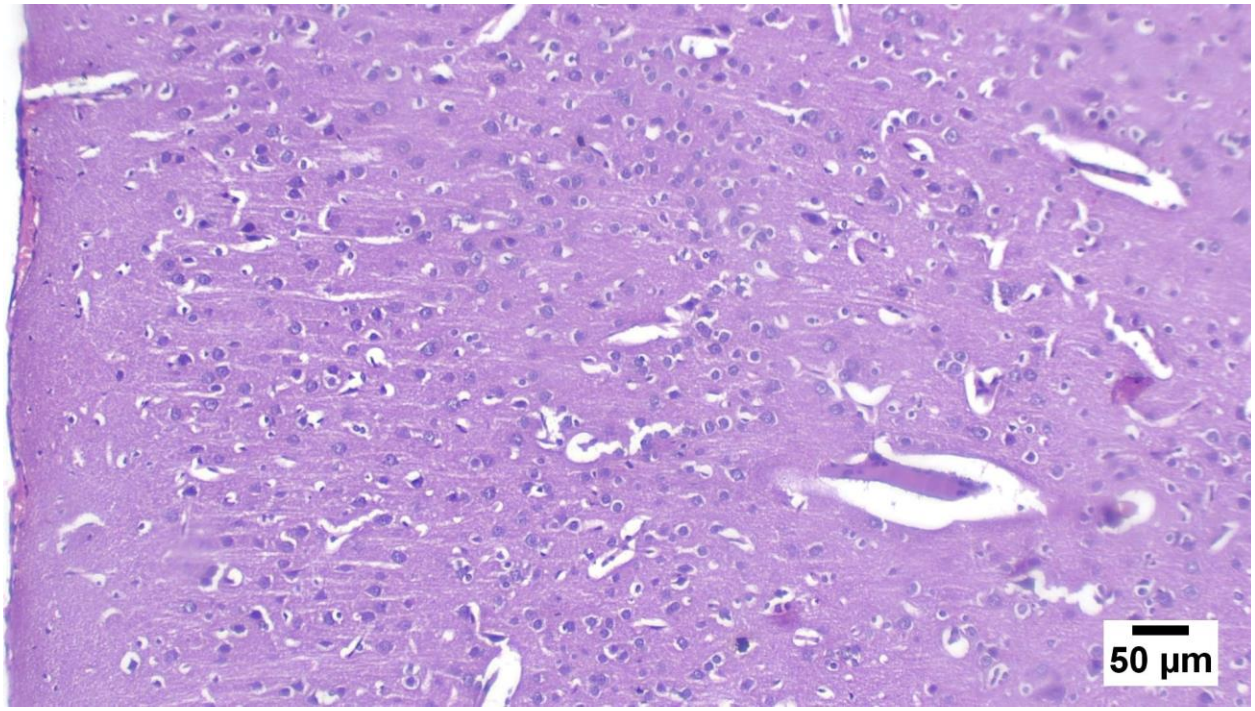

Photomicrograph of brain cerebral cortex of group 3 showing apparently normal cerebral cortex (H&E).

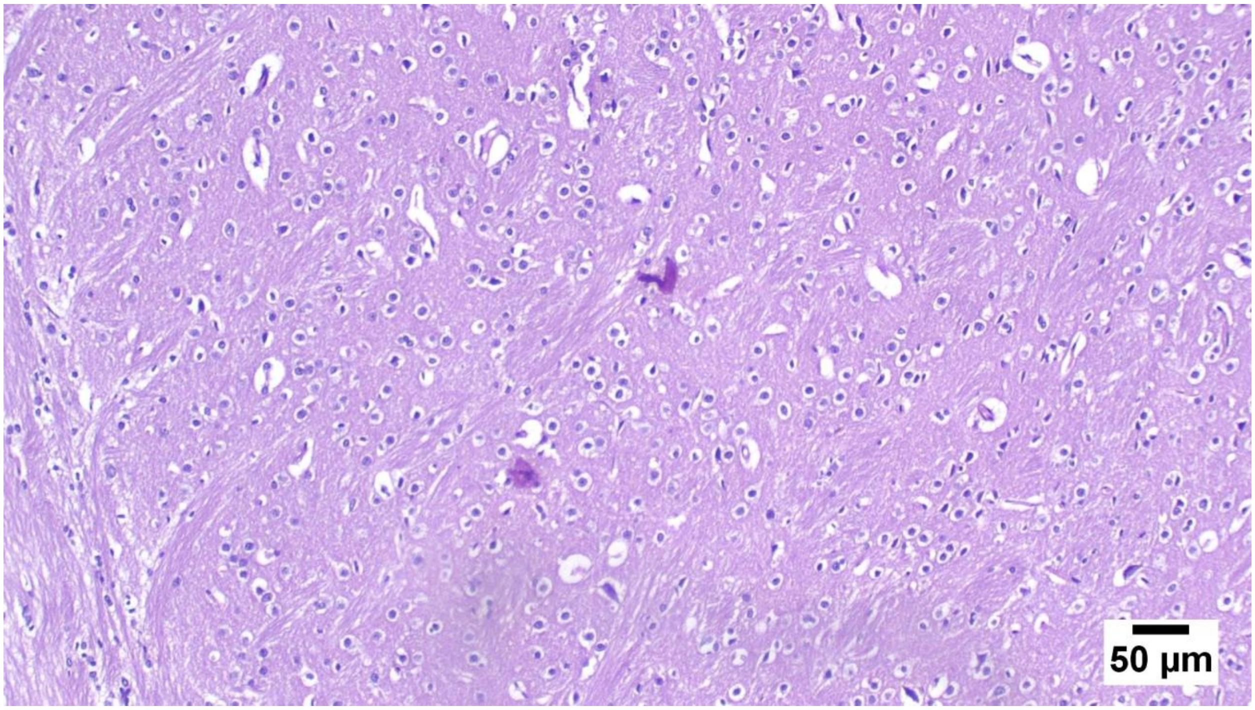

Photomicrograph of brain striatum of group 3 showing mild neuronal edema (H&E).

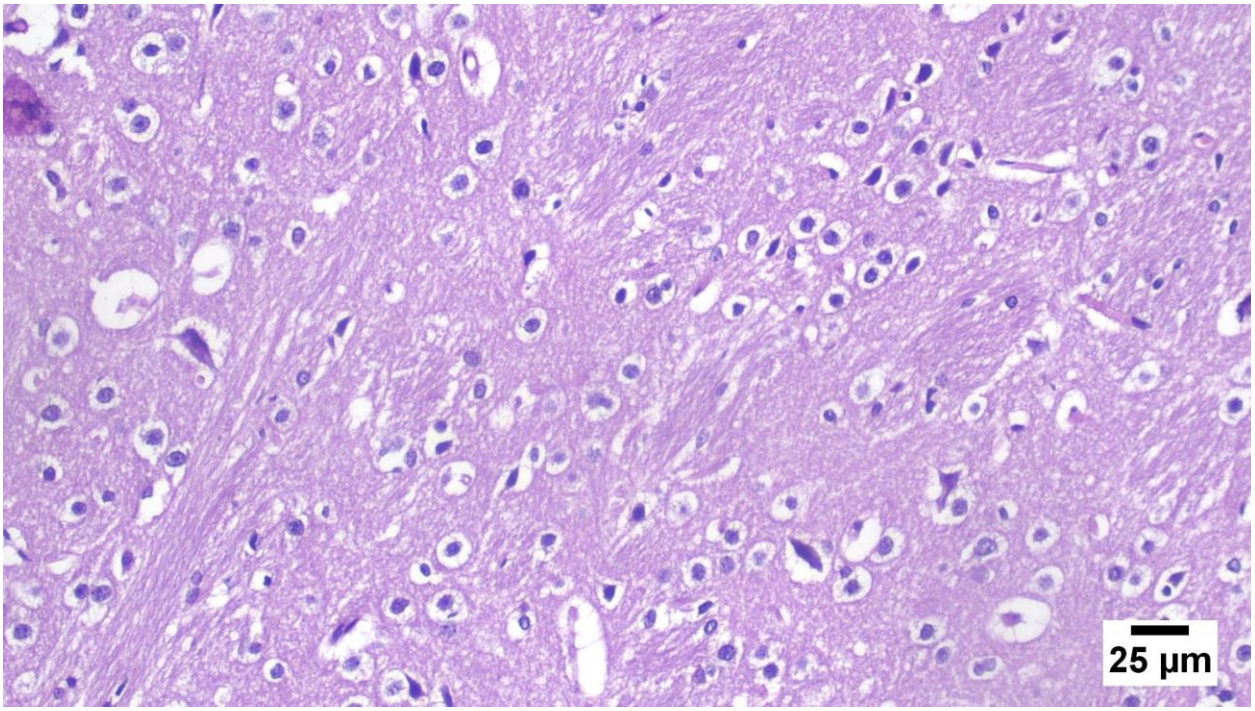

Photomicrograph of brain striatum of group 3 showed higher magnification showing mild neuronal edema (H&E).
