# Supplementary material for: In vivo study of dose-dependent antioxidant efficacy of functionalized core–shell yttrium oxide nanoparticles
Source: Naunyn Schmiedebergs Arch Pharmacol. 2022 Feb 24;395(5):593–606. doi: 10.1007/s00210-022-02219-1 (PMC8989852; doi:10.1007/s00210-022-02219-1)
Supplement: Supplementary file 2 — Supplementary file2 (PDF 1999 KB) [file 210_2022_2219_MOESM2_ESM.pdf]

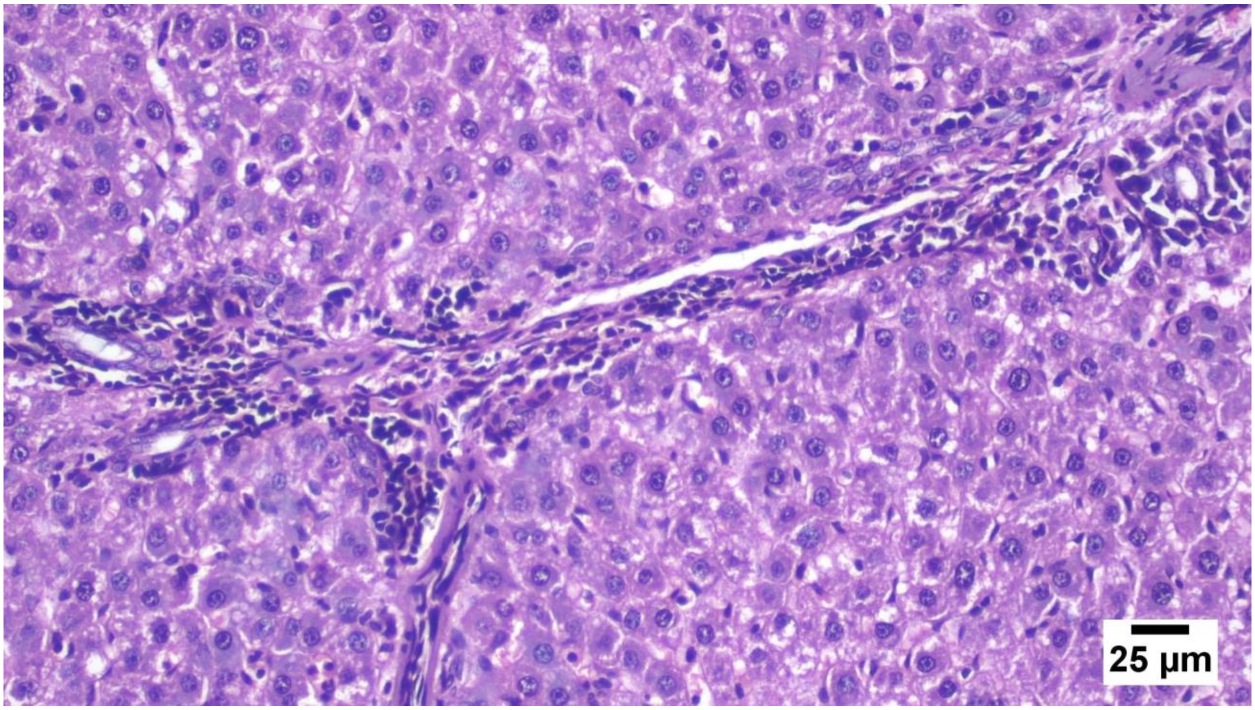

Photomicrograph of liver, group 1 showing portal hepatitis (H&E).

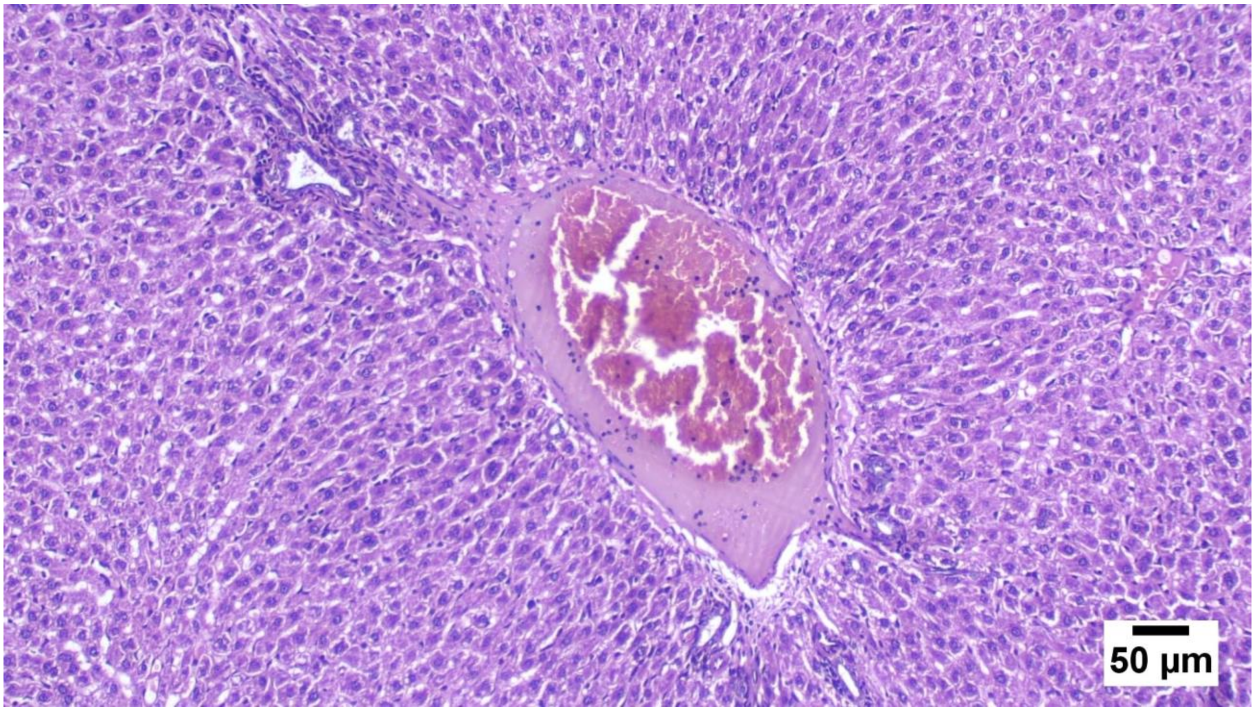

Photomicrograph of liver, group 1 showing congestion of portal edema (H&E).

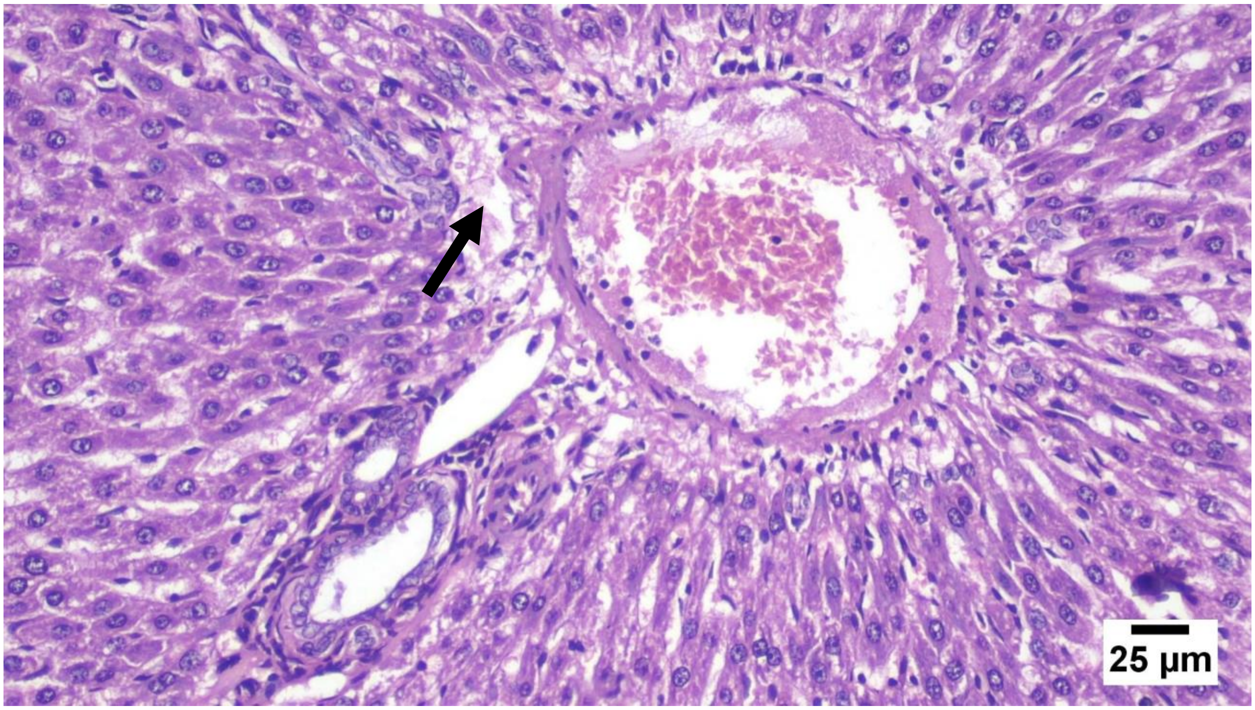

Photomicrograph of liver, group 1 showing perivascular edema in the portal blood vessel (arrow) (H&E).

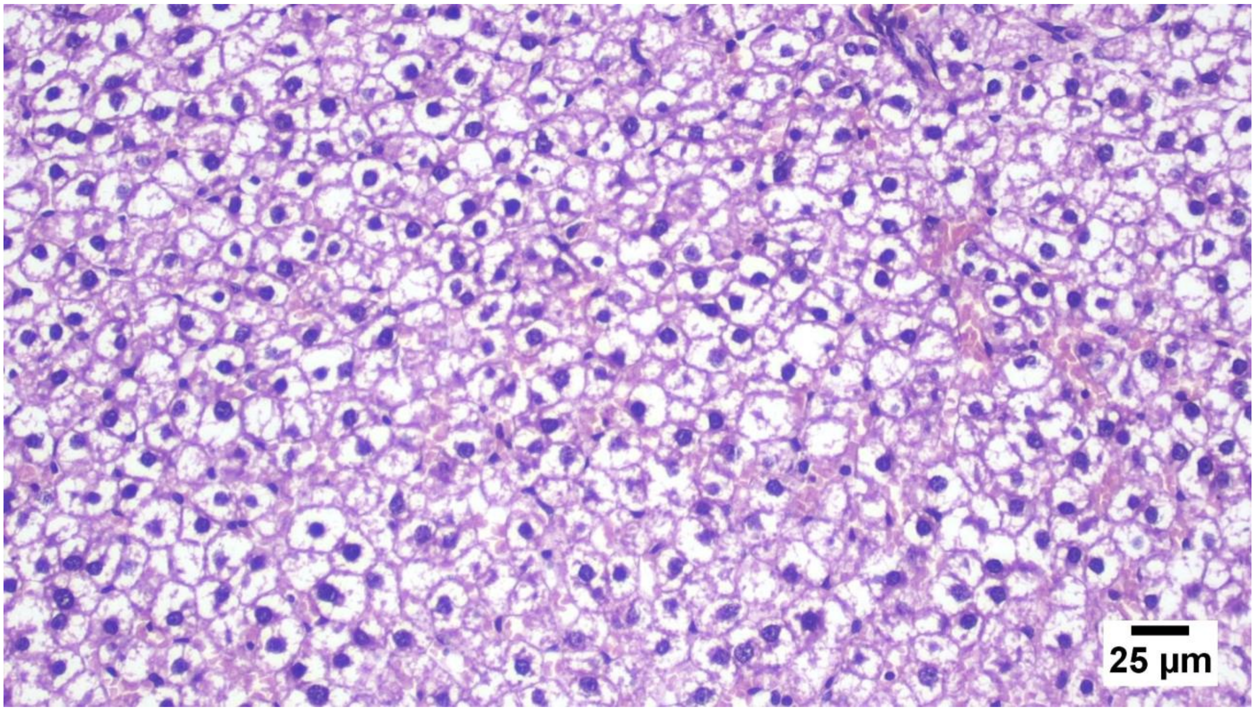

Photomicrograph of liver, group 2 showing vacuolated hepatic parenchyma (H&E).

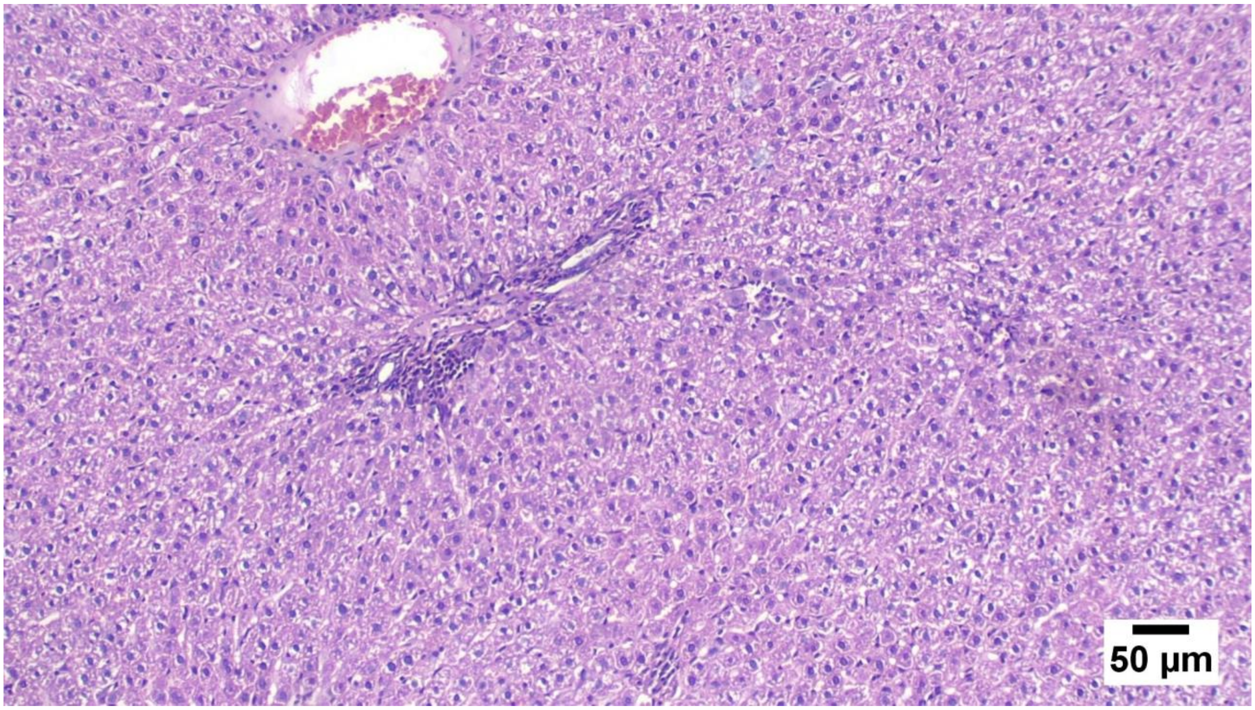

Photomicrograph of liver, group 2 showing few mononuclear cells infiltrating the portal triad (H&E).

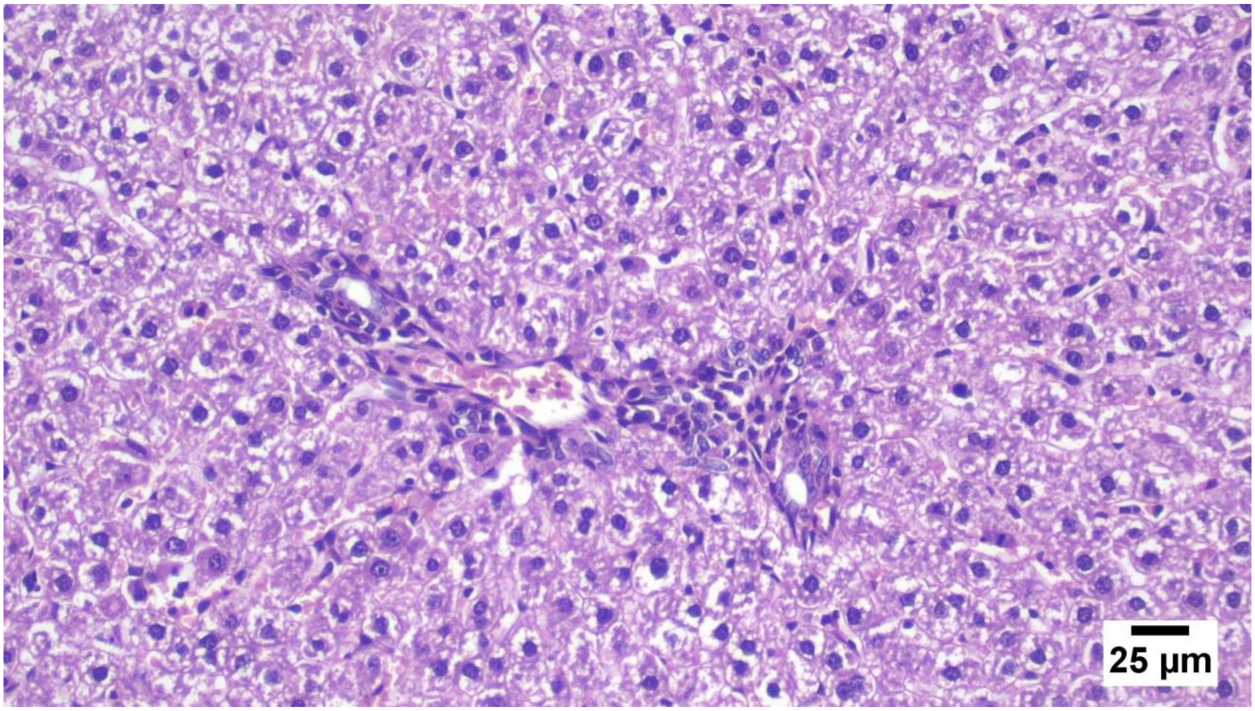

Photomicrograph of liver, group 2 higher magnification showing mononuclear inflammatory cells accumulated in the portal area (H&E).

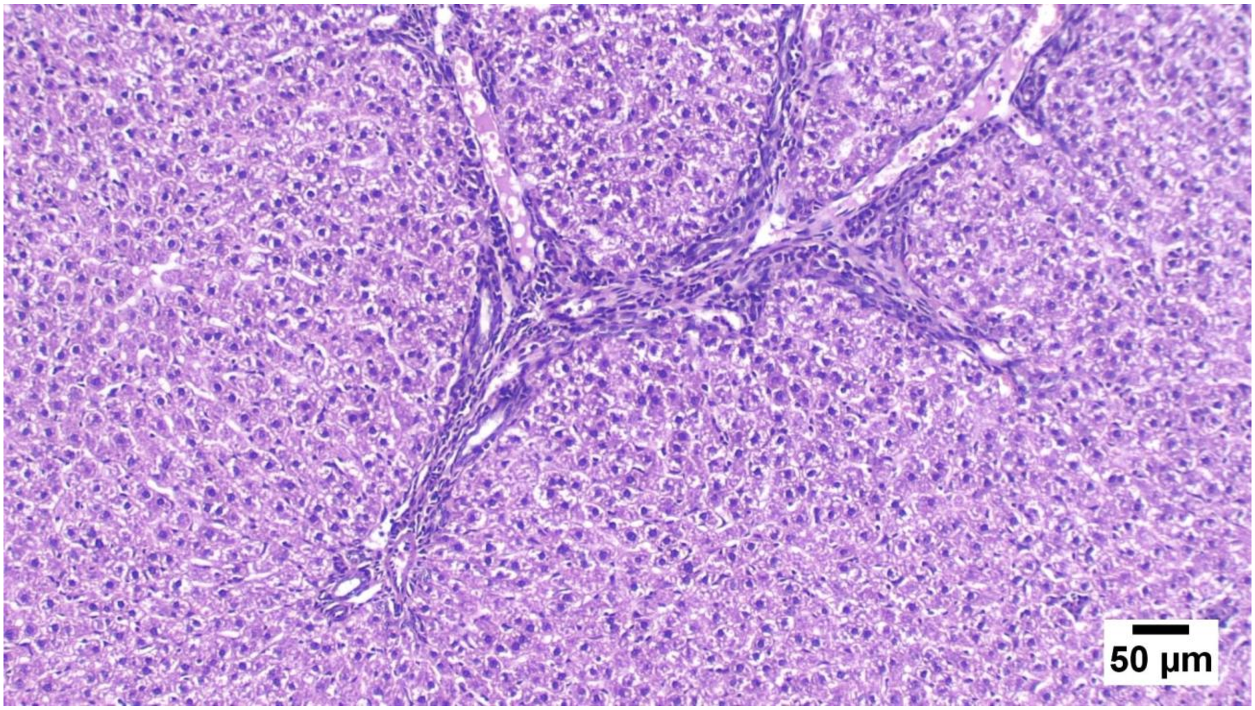

Photomicrograph of liver, group 2 showing mild portal fibroplasia with inflammatory cells infiltration (H&E).

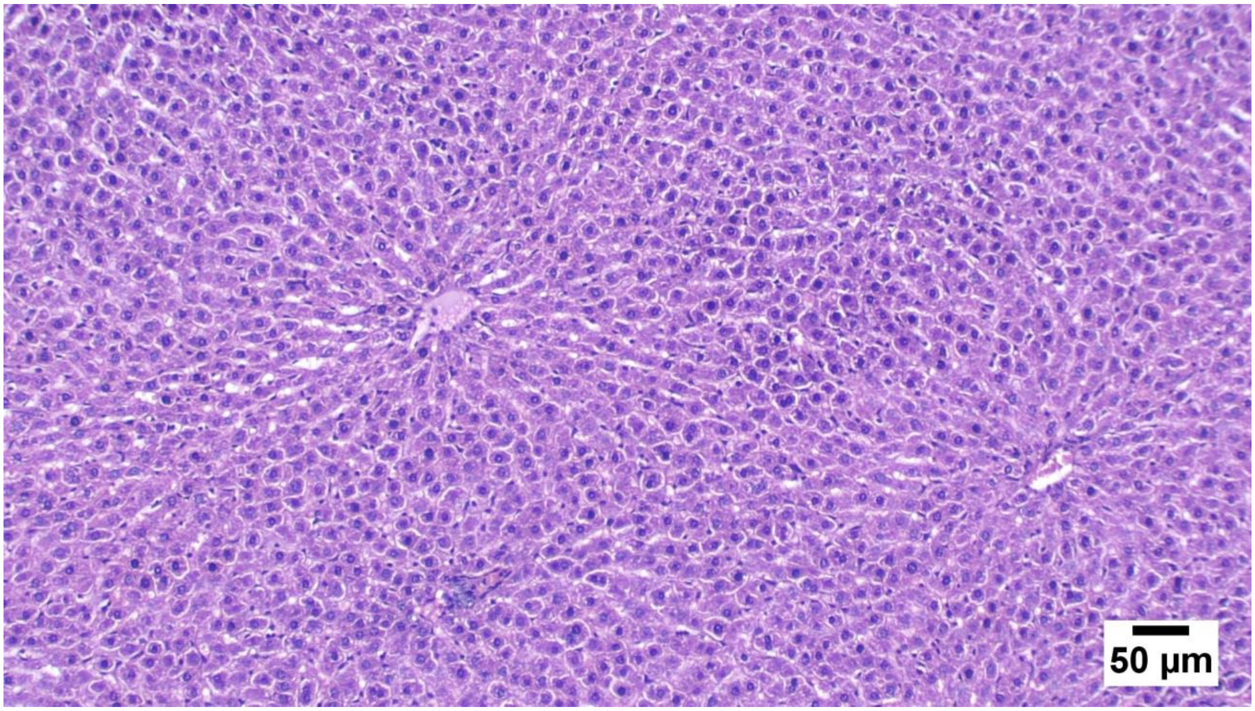

Photomicrograph of liver, group 3 showing apparently normal hepatic parenchyma (H&E).

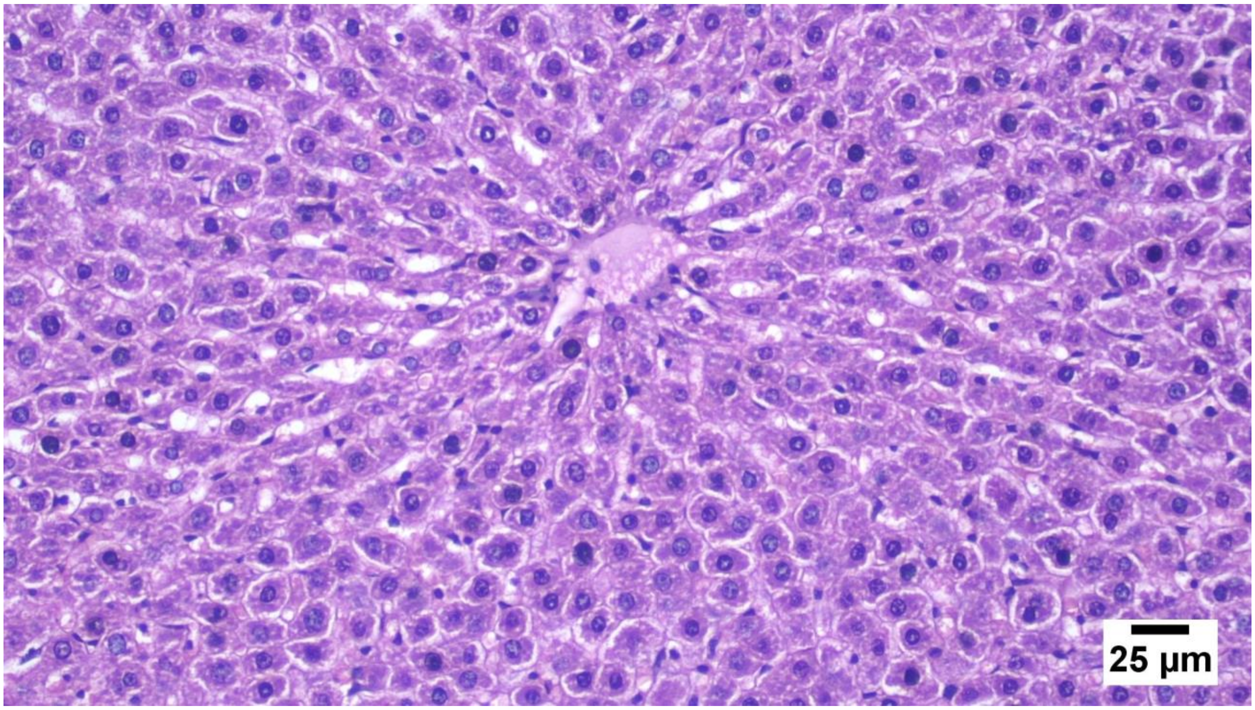

Photomicrograph of liver, group 3 showing apparently normal hepatocytes surrounding the central vein (H&E).
