# Supplementary material for: In vivo study of dose-dependent antioxidant efficacy of functionalized core–shell yttrium oxide nanoparticles
Source: Naunyn Schmiedebergs Arch Pharmacol. 2022 Feb 24;395(5):593–606. doi: 10.1007/s00210-022-02219-1 (PMC8989852; doi:10.1007/s00210-022-02219-1)
Supplement: Supplementary file 3 — Supplementary file3 (PDF 3210 KB) [file 210_2022_2219_MOESM3_ESM.pdf]

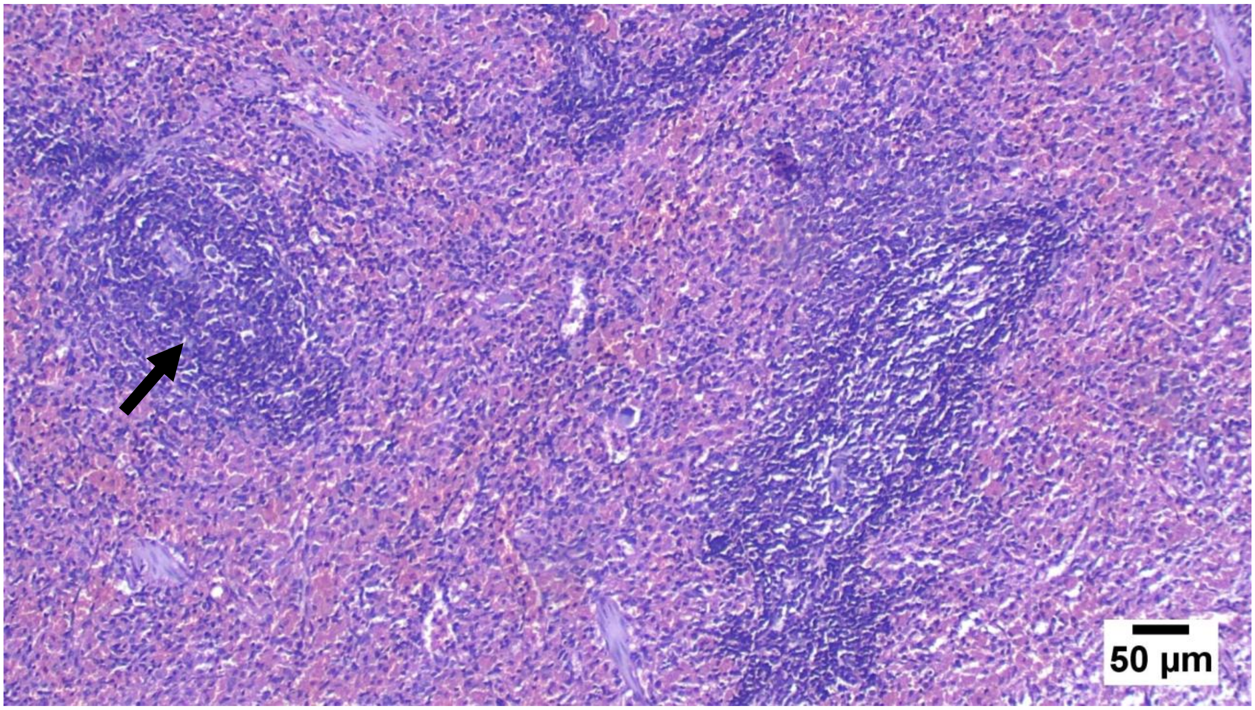

Photomicrograph of spleen, group 1 showing relatively small lymphoid follicle (arrow) with expansion of the red pulp (H&E).

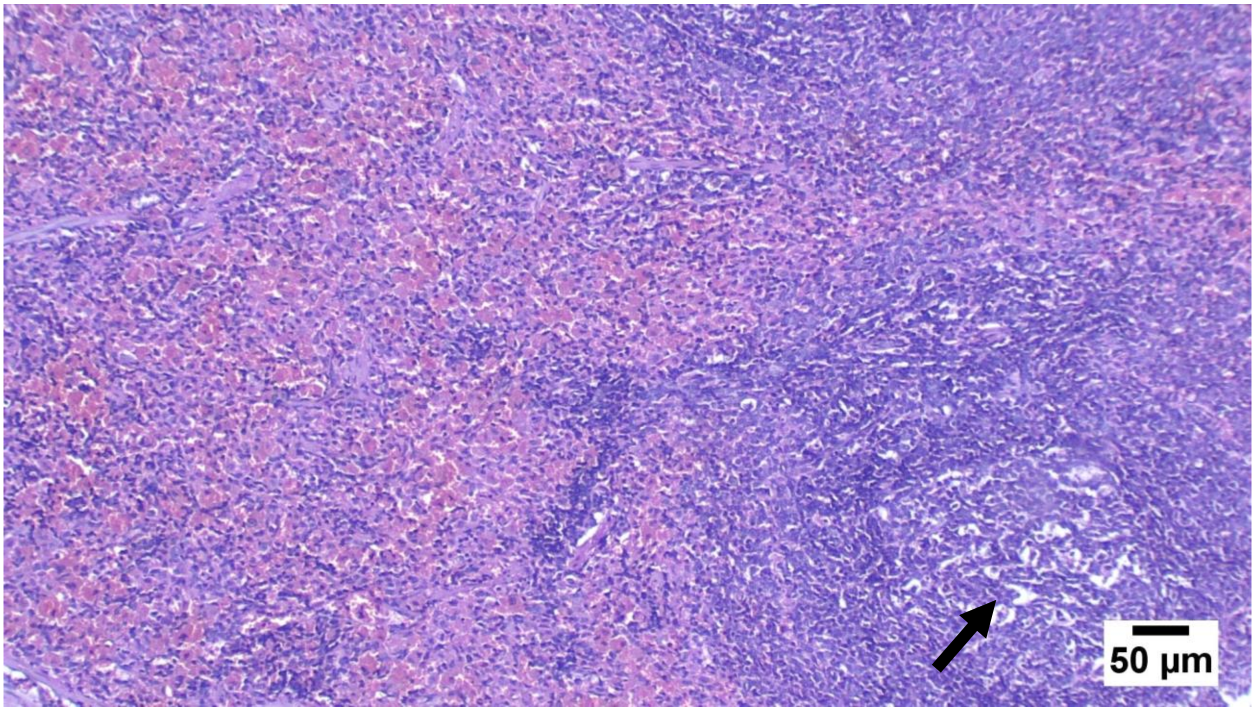

Photomicrograph of spleen, group 1 showing relatively small lymphoid follicle with depletion (arrow) and expansion of the red pulp (H&E).

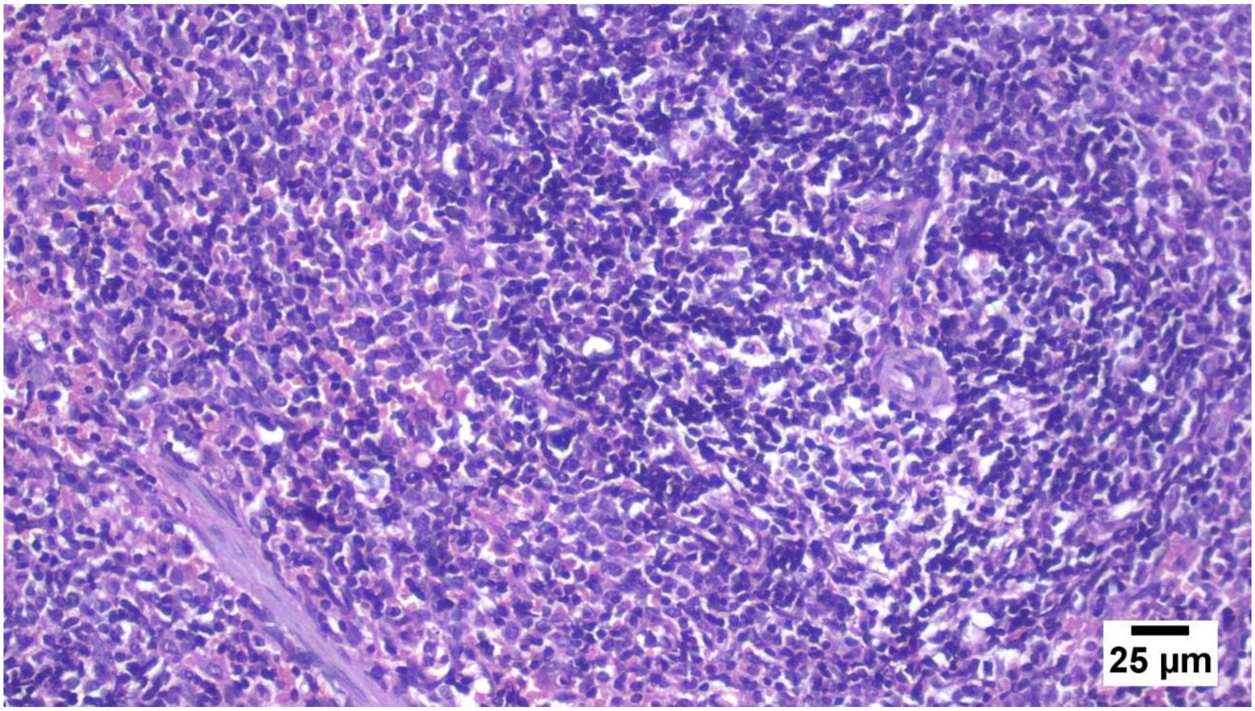

Photomicrograph of spleen, group 1 showing lymphoid depletion (H&E).

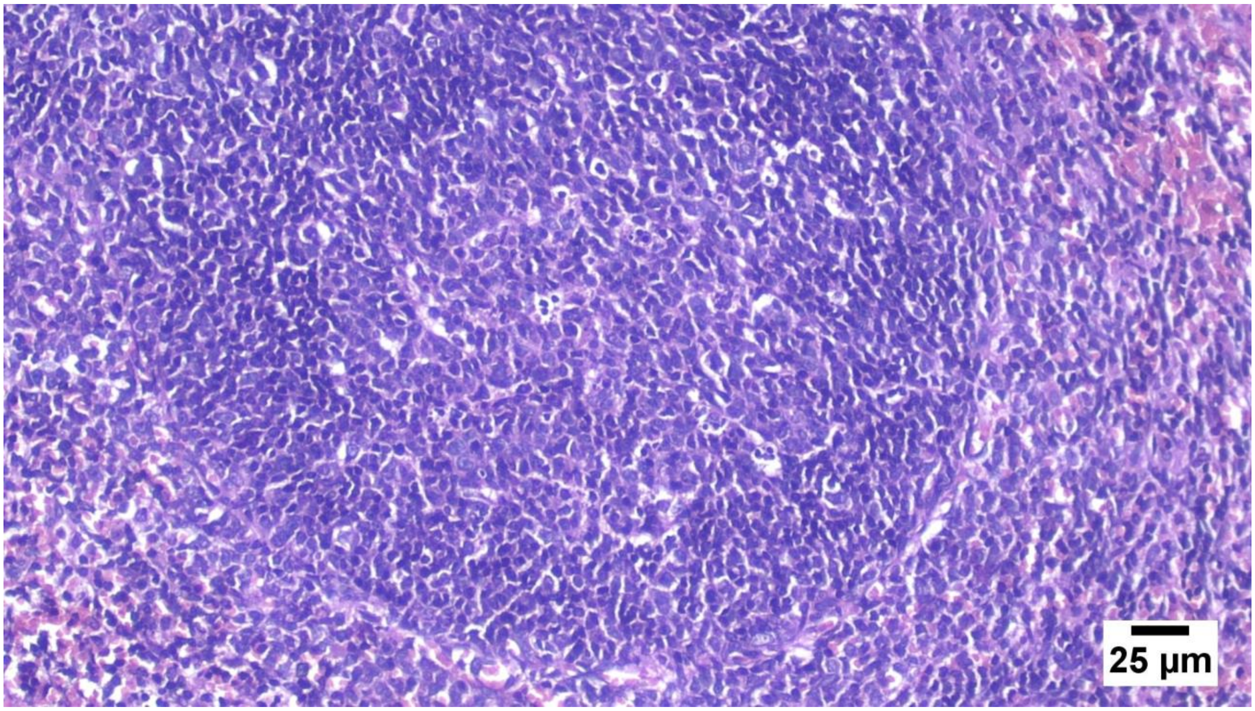

Photomicrograph of spleen, group 2 higher magnification showing apparently normal lymphoid follicle (H&E).

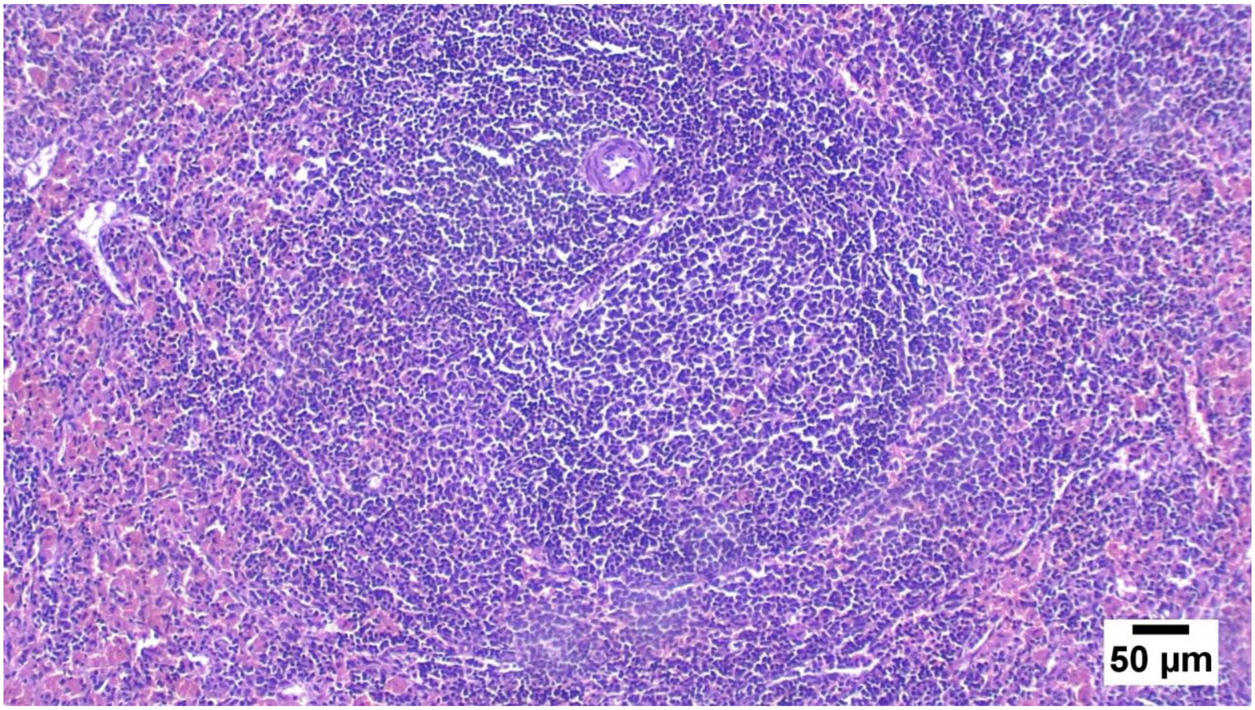

Photomicrograph of spleen, group 2 showing apparently normal splenic tissue (H&E).

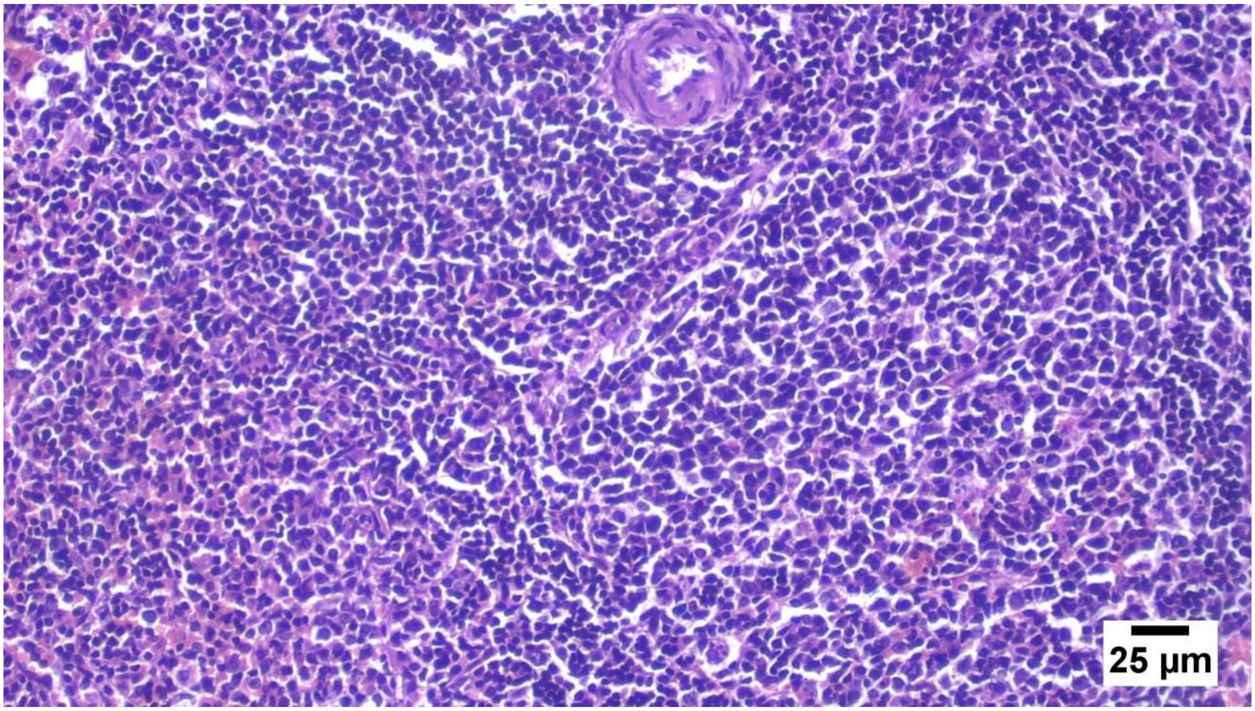

Photomicrograph of spleen, group 2 higher magnification showing apparently normal lymphoid follicle (H&E).

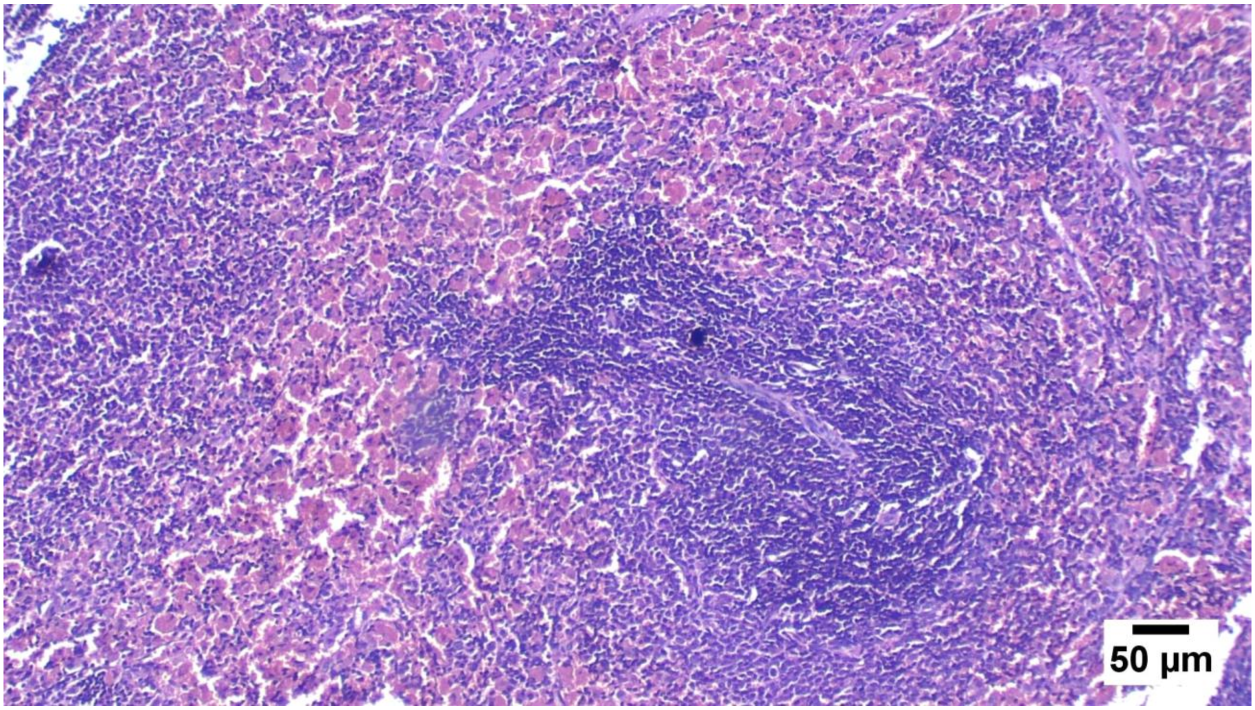

Photomicrograph of spleen, group 2 showing apparently normal splenic tissue (H&E).

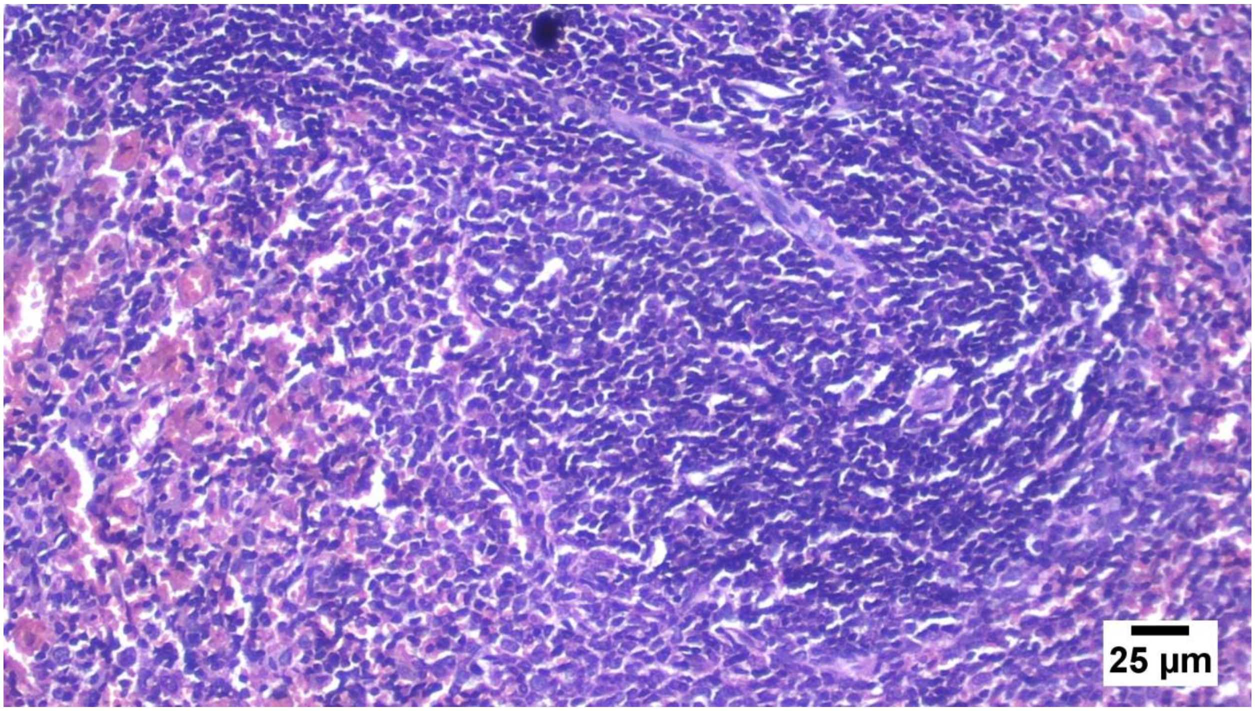

Photomicrograph of spleen, group 2 higher magnification showing apparently normal lymphoid follicle (H&E).

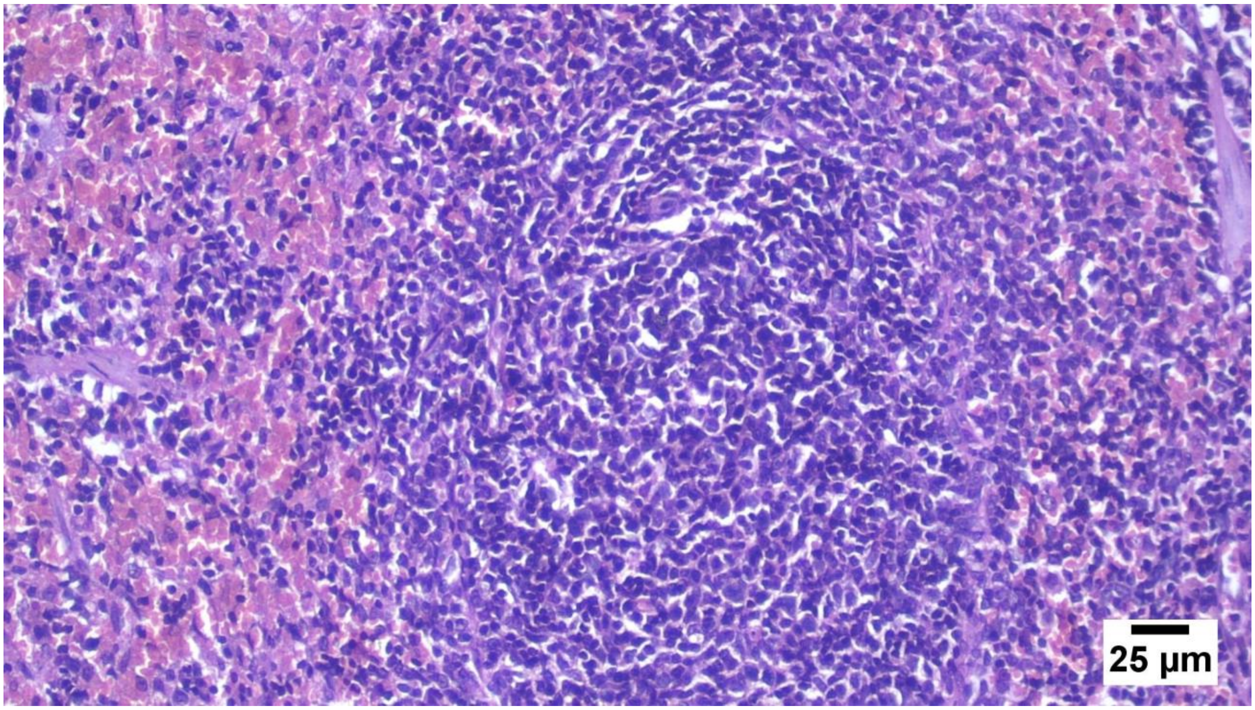

Photomicrograph of spleen, group 3 higher magnification showing apparently normal lymphoid follicle (H&E).

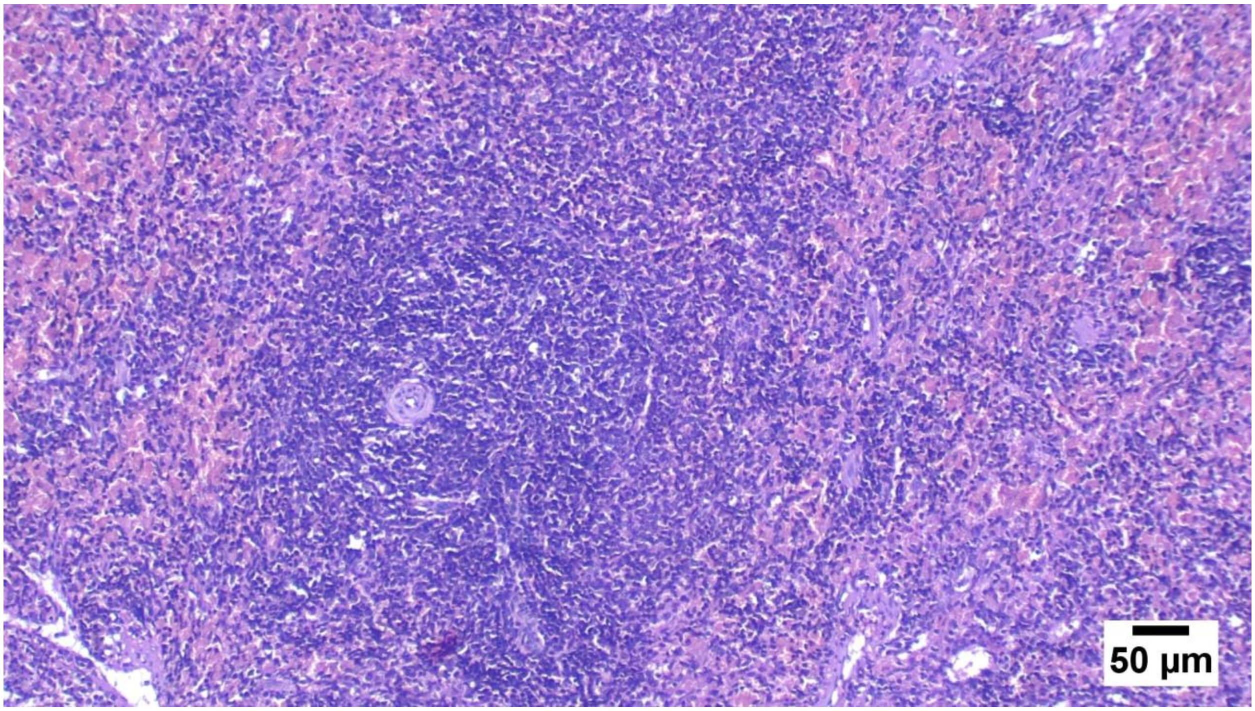

Photomicrograph of spleen, group 3 showing apparently normal splenic tissue (H&E).

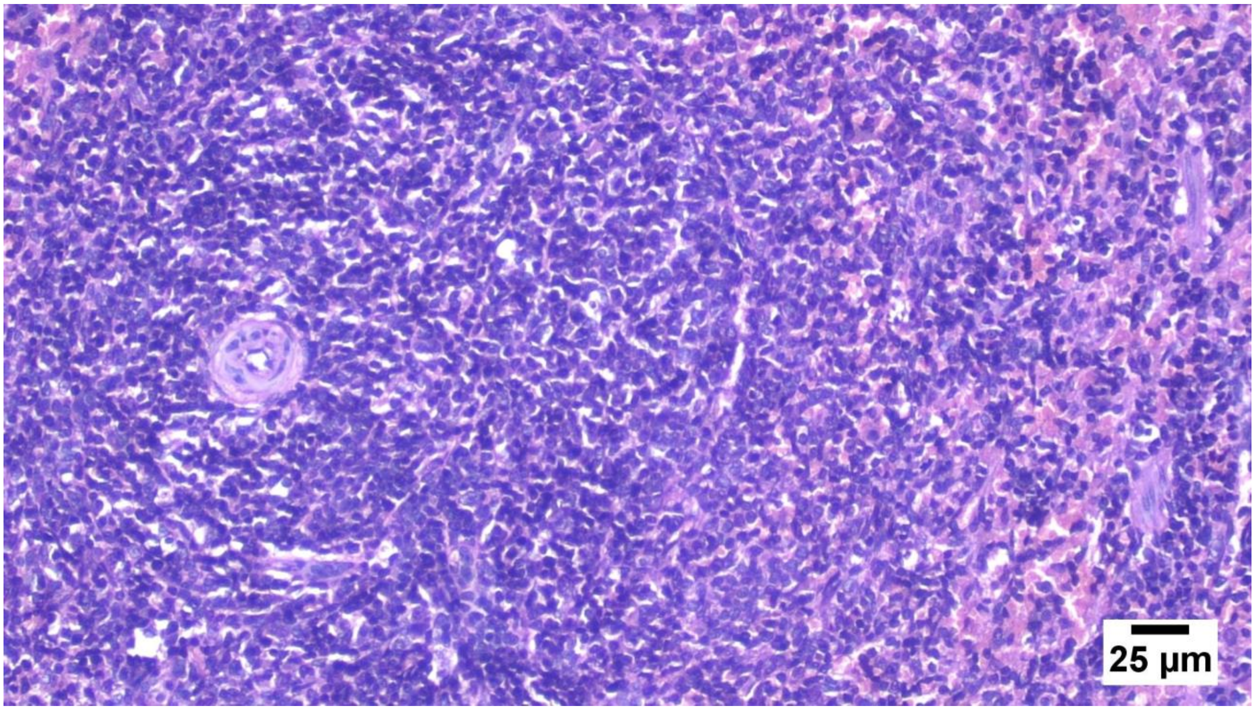

Photomicrograph of spleen, group 3 higher magnification showing apparently normal lymphoid follicle (H&E).

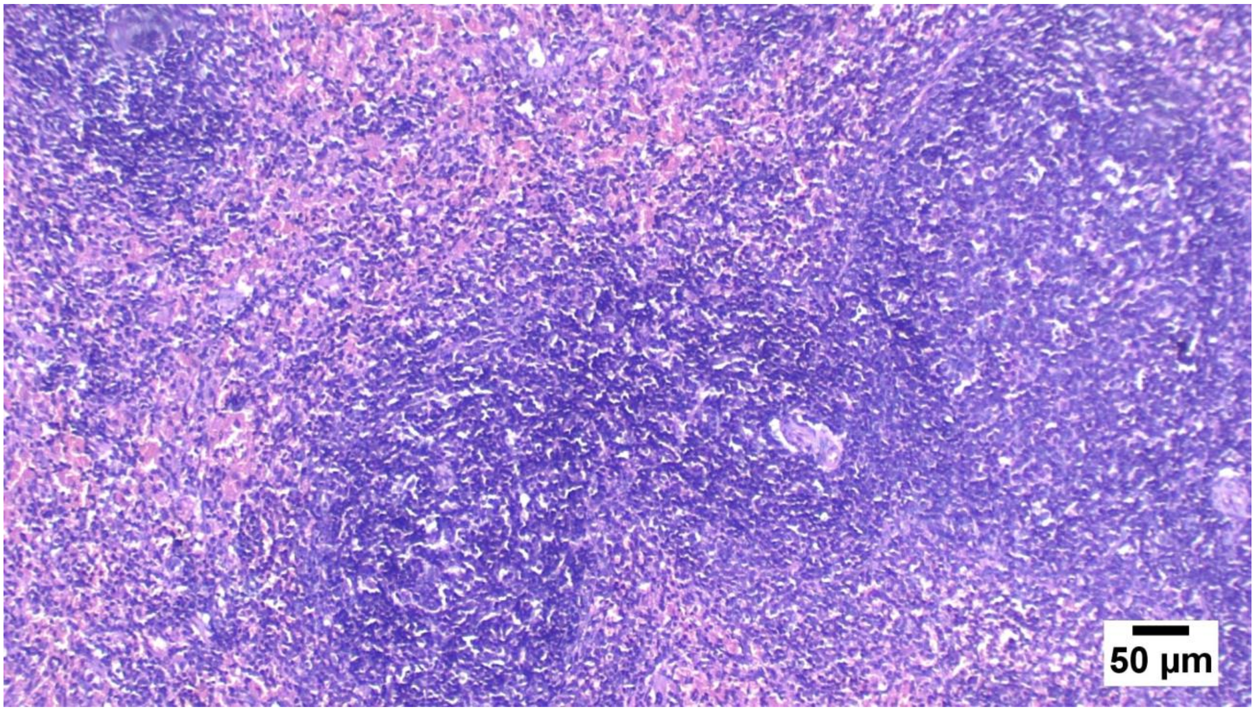

Photomicrograph of spleen, group 3 showing apparently normal splenic tissue (H&E).

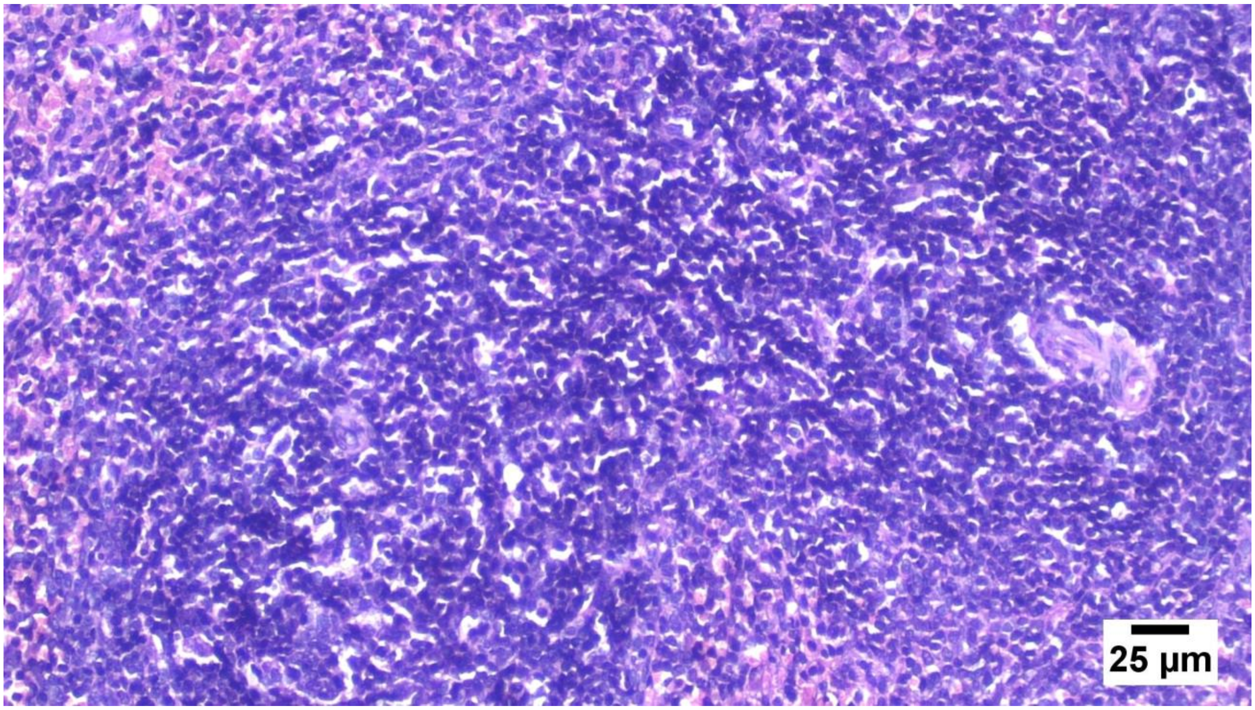

Photomicrograph of spleen, group 3 higher magnification showing apparently normal lymphoid follicle (H&E).
